# Benchmarking 3D-ΔPDF analysis using in-house X-ray sources

**DOI:** 10.1107/S2053273325003389

**Published:** 2025-05-19

**Authors:** Karl O. R. Juul, Kristoffer A. H. Støckler, Bo B. Iversen

**Affiliations:** a Center for Integrated Materials Research, Department of Chemistry and iNANO Aarhus University Aarhus DK-8000 Denmark; University of Warsaw, Poland

**Keywords:** 3D-ΔPDF, diffuse scattering, correlated disorder, pair-distribution function

## Abstract

New detector technology has in recent years improved the data quality available from in-house X-ray diffractometers. A recent study compared high-resolution low-temperature X-ray diffraction data obtained from modern in-house diffractometers with synchrotron data in relation to extracting subtle electron-density details using the multipole model [Vosegaard *et al.* (2023). *Acta Cryst.* B**79**, 380–391]. It was concluded that for organic molecular crystals excellent agreement can be obtained, and only subtle electron-density details are better resolved at the synchrotron sources. This study aims to benchmark the quality of weak diffuse scattering data and three-dimensional difference pair-distribution function (3D-ΔPDF) analysis for in-house X-ray sources against more accurate and better resolved synchrotron data using three examples (Cu_1.95_Se, Nb_1−*x*_CoSb and InTe). Since the 3D-ΔPDF method is still relatively new in crystallographic research, we also provide a general description of the pipeline of analysis. The three selected systems highlight important differences in correlated disorder and the corresponding analysis. In all three cases, the synchrotron data have better signal-to-noise ratios and extend to higher scattering vectors. Using the in-house 3D-ΔPDF on Cu_1.95_Se, the same ordered 2D superstructure can be determined as for the synchrotron data, although additional arguments based on order within a 2D supercell or on ionic radii must be used to obtain an adequate model. For Nb_1−*x*_CoSb, the preference for vacancies to avoid each other and the size effect associated with structural relaxation of the lattice near vacancies can also be observed and assigned in the in-house 3D-ΔPDF. For InTe, the weak diffuse scattering, radial broadening and higher temperature than the original study mean that, although most of the important features are visible in the in-house data, some features are obscured, and the full correlated disorder model cannot be constructed. Overall, it is found that many of the conclusions derived from synchrotron data can also be extracted from in-house data, but in some cases additional postulates are needed, and in general subtle details may be too noisy to be properly interpreted in the in-house data.

## Abstract 2

Measurement of diffuse scattering data and subsequent three-dimensional difference pair-distribution function (3D-ΔPDF) analysis of correlated disorder are compared for in-house and synchrotron sources.

## 1. Introduction

For many functional materials, their properties arise from defects in the structure. In some cases, these properties may be significantly different from what can be obtained in ordered structures (Simonov & Goodwin, 2020 ▸). This is especially evident in thermoelectric materials where defects are often found to be the origin of the low thermal conductivity desired for these materials (Zhang *et al.*, 2021 ▸; Roth & Iversen, 2019 ▸; Roth *et al.*, 2021 ▸; Snyder & Toberer, 2008 ▸; Sangiorgio *et al.*, 2018 ▸; Holm *et al.*, 2020 ▸). Often, the disorder is not random but correlated in some way. A classic example is the orientation of water molecules in ice (Pauling, 1935 ▸). Correlations within the disorder have been shown to allow for greater flexibility when, *e.g.*, engineering electronic bands (Roth & Goodwin, 2023 ▸), designing metal–organic frameworks for information storage (Ehrling *et al.*, 2021 ▸; Meekel *et al.*, 2023 ▸), enhancing properties of battery materials (Simonov *et al.*, 2020 ▸), or in the aforementioned thermoelectric materials. Such structural disorder may be analysed using the three-dimensional difference pair-distribution function (3D-ΔPDF) obtained by Fourier transforming full 3D volumes of X-ray (Koch *et al.*, 2021 ▸; Davenport *et al.*, 2021 ▸; Schmidt, Neder *et al.*, 2023 ▸; Urban *et al.*, 2015 ▸; Simonov *et al.*, 2014 ▸), neutron (Schmidt, Neder *et al.*, 2023 ▸) and recently also electron diffuse scattering (Schmidt, Klar *et al.*, 2023 ▸; Poppe *et al.*, 2024 ▸).

The 3D-ΔPDF method is based on the same principles as the normal Patterson function, but the features of the 3D-ΔPDF show how the real structure of a disordered material deviates from its average structure. This, of course, builds on the assumption that the average structure is well known, and throughout this work, it will be assumed that this is the case. The total intensity of the scattered beam can be described as 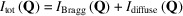. The first term describes the average structure. The Patterson function used for average structure solution is 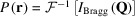. The total three-dimensional pair-distribution function (3D-PDF) is defined as PDF(**r**) = 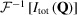. If the average structure is known, the local structure can be analysed by examining the deviations from the average structure. For this, the 3D-ΔPDF is defined as 3D-ΔPDF(**r**) = 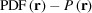 (Weber & Simonov, 2012 ▸). It can be calculated as
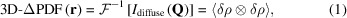
where 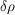 is the difference in electron density, 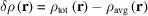, 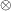 is the cross-correlation operator, and the average 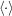 is the experimental time average (Roth & Iversen, 2019 ▸). Unlike the 3D-PDF, features in the 3D-ΔPDF can be negative (Koch *et al.*, 2021 ▸; Weber & Simonov, 2012 ▸). It is possible to calculate the sign of a peak using
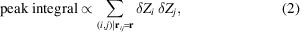
where 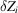 is the difference in the number of electrons at the *i*th site between the real and average structure and the sum is over all sites separated by the same interatomic vector (Roth & Iversen, 2019 ▸). Equation (2) allows negative peak integrals if, *e.g.*, one of the sites tends to have fewer electrons than the average while the other tends to have more electrons. Such correlations are referred to as negative correlations (Weber & Simonov, 2012 ▸).

Some of the advantages of the 3D-ΔPDF compared with the 3D-PDF are that the integral errors on the Bragg intensities are of the same magnitude (or larger) than the integral intensities of the diffuse scattering. Thus, removing the Bragg peaks significantly enhances the quality of the 3D-ΔPDF compared with the 3D-PDF when analysing the local structure. The 3D-PDF is also heavily dominated by the average structure correlations. Removing those gives higher contrast in the 3D-ΔPDF which allows qualitative and/or semi-quantitative conclusions based purely on visual inspection (Weber & Simonov, 2012 ▸).

The 3D-ΔPDF method is unique to single crystals as the orientational average inherent to powder diffraction collapses the Bragg and diffuse scattering onto a 1D line from which it becomes very difficult to separate Bragg and diffuse intensities in the powder pattern (Weber & Simonov, 2012 ▸). Furthermore, the directional information is lost as a consequence of the orientational averaging.

Due to the weakness of the diffuse scattering signal compared with the Bragg scattering, studies have previously been made at synchrotron facilities due to the high intensity of the X-rays and high photon energy available. Vosegaard *et al.* (2023 ▸) showed that only the subtle electron-density details were better resolved on synchrotron sources. The recent implementations of hybrid pixel photon-counting detectors (Förster *et al.*, 2019 ▸) on in-house diffractometers have enabled studies of diffuse scattering and correlated disorder using the 3D-ΔPDF method on data collected using such instruments (Meekel *et al.*, 2023 ▸; Schmidt, Klar *et al.*, 2023 ▸; Schmidt, Neder *et al.*, 2023 ▸). The ability to measure diffuse scattering in-house makes the method more flexible as applications for beamtime and travel can be avoided. This is also favourable due to the comparative cheapness of in-house sources, and in cases where synchrotron facilities may be inaccessible due to long waiting time, upgrade programs or pandemics. However, in order for the in-house 3D-ΔPDF to become a well established technique, it is necessary to compare the data quality with that obtainable using higher-resolution synchrotron-based scattering.

In this study, we aim to benchmark the in-house 3D-ΔPDF method by comparing the in-house 3D-ΔPDF from three crystals with different types of disorder and diffuse scattering with synchrotron measurements conducted on the same materials and by performing the same analysis as in previous publications using the synchrotron data (Roth *et al.*, 2021 ▸; Roth & Iversen, 2019 ▸; Støckler, Zhang *et al.*, 2024 ▸). To ensure proper representation of each source, the data were collected and reduced to show the full capabilities of the source within a reasonable measuring time. Furthermore, the in-house measurements have been conducted at the same temperatures with the exact same crystal specimens as were used for the synchrotron measurements (Cu_1.95_Se, Nb_1−*x*_CoSb and InTe). For InTe, additional synchrotron data were collected at 100 K, the lowest temperature possible with the cryostat mounted on the in-house instrument [compared with 25 K used in the original study by Støckler, Zhang *et al.* (2024 ▸)]. The crystal systems have been chosen to highlight three different aspects of diffuse scattering. First, Cu_1.95_Se with strong diffuse scattering away from the Bragg peaks is chosen to highlight the principles of the method. Second, Nb_1−*x*_CoSb half-Heuslers (HH) are examined due to their weaker diffuse scattering located away from the Bragg peaks to probe the sensitivity of the method. Lastly, the limitations of the method are tested with InTe which gives rise to weak diffuse scattering overlapping with the Bragg peaks.

We expect the differences between the in-house and synchrotron methods to be a lower resolution due to lower-energy X-rays on the in-house diffractometer compared with the synchrotron, lower signal-to-noise ratio, as well as the polychromic nature of X-ray sources using both *K*α_1_ and *K*α_2_ radiation, which will give rise to radially broadened peaks. These should, respectively, lead to a lower-resolution 3D-ΔPDF, more noise in the 3D-ΔPDF and larger punched volumes during data reduction, leading to increased danger of punching diffuse scattering along with the Bragg peaks.

## 2. Technical aspects of the 3D-ΔPDF method

The workflow required for generating an experimental 3D-ΔPDF is summarized in detail by Koch *et al.* (2021 ▸); however, a simplified overview is given here to highlight the most important points. In general, the workflow can be split into five parts.

### 2.1. Data acquisition

Before the data acquisition, a suitable high-quality single crystal must be identified. As for the case of normal crystallographic measurements, high quality in this case means that the crystal is of low mosaicity and, if possible, has a fairly isotropic crystal shape to limit anisotropic absorption effects. The crystal should also be small to limit general effects of absorption – as a rule of thumb, crystal dimensions of 50–100 µm offer a good compromise, since the diffuse scattering signal will of course also be weaker for smaller crystals. Various ways of mounting the sample are possible; however, all the data presented here were collected on samples mounted on a thin (∼10 µm) glass capillary using a small amount of ep­oxy glue. Practically, the glue is mixed, and a small droplet is placed on a glass slide. A crystal is then fully submerged in the glue and the glass capillary is used to push the crystal out of the glue droplet. By pushing the now glue-covered crystal across the glass slide using the capillary excess glue is deposited on the slide leaving only a thin layer on the crystal. The crystal is then gently picked up, preferably using the very tip of the glass capillary, and the sample is ready for data collection as soon as the ep­oxy glue has cured, which can be evaluated by the remaining glue left on the glass slide.

The source is generally chosen based on optimizing the scattered intensity and 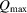. From elemental X-ray sources, a higher atomic number of the source leads to lower wavelength (from Moseley’s law) which in turn leads to higher 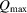 (which is a measure of the 3D-ΔPDF resolution as described in the section on Fourier transformation below). However, the scattered intensity is proportional to 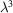 (Giacovazzo *et al.*, 2011 ▸), and thus the higher attainable 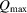 possible with shorter-wavelength sources comes at a cost of lower scattered intensities. Furthermore, the flux of the characteristic X-rays varies across the different sources.

Before starting a data collection, it is also important to ensure that the chosen wavelength is compatible with the elemental composition of the crystal. Fluorescence is particularly detrimental to 3D-ΔPDF analysis since it is independent of the X-ray polarization – this means that a highly structured background is introduced when applying the pixel-wise polarization correction during the data reduction. As illustrated in the supporting information for Cu_2_Se, this is an issue when using a molybdenum source even though the X-ray energy is almost 5 keV above the *K* edge of selenium. In such cases it is particularly helpful to be able to change the energy threshold of the detector to an energy higher than the energy of the emitted fluorescent X-rays.

The diffuse scattering data collection itself is performed in a similar manner to an ordinary single-crystal experiment (examples of data collection strategies for both in-house and synchrotron measurements are given in the supporting information) although possibly with longer exposure times to increase counting statistics of the weak diffuse scattering. In general, full completeness up to a scattering angle as high as possible is needed to produce a 3D-ΔPDF. For high-symmetry crystals this may be achieved from very few runs whereas lower-symmetry crystals require the measurement of multiple runs at different κ (or χ) angles to increase data completeness. Commercial software can in most cases give estimates for completeness on in-house instruments while this is often not the case on synchrotron sources – however, in the latter case, the brightness of the source allows for measuring many redundant runs in a short timeframe, and thus this is not much of an issue in practice.

After collecting the diffuse scattering data, a background measurement should be performed. In our experiments we generally choose to measure only the air scattering, *i.e.* we retract the crystal from the beam without moving the beamstop and measure around 100 frames of air scattering per detector position (examples of air scattering data collection strategies are available in the supporting information). The air scattering for each detector position is then averaged, giving a single averaged air scattering frame for each detector position. It is worth noting that using air scattering as the experimental background has its limitations. For example, it does not account for background arising from scattering from the glass capillary or the ep­oxy in the beam. Trying to minimize the amount of ep­oxy used and mounting the crystal at the very tip of the capillary serve to mitigate these issues. Another way to minimize these scattering contributions would be to use smaller beam sizes which could be an option at synchrotron sources. However, in our measurements the beam sizes at the synchrotron measurements [∼200 × 200 µm at the Advanced Photon Source (APS), 15-ID-D, ∼130 × 145 µm at SPring-8, BL02B1] were comparable to the beam size on our in-house instrument (∼140 × 140 µm). Another issue is that the air scattering measured without a sample in the beam will be overestimated due to the lack of beam attenuation by the sample. This can result in negative scattering intensity in the background-subtracted data at low angle but can be relatively easily identified by visual inspection. An *adhoc* solution for this problem is to include a scale factor smaller than one for the background during the subtraction.

### 2.2. Data reconstruction

To produce a 3D-ΔPDF from the raw scattering data, the scattering must be reconstructed in 3D reciprocal space. To make the subsequent steps of symmetrization and punching of Bragg peaks more straightforward, it is helpful to reconstruct the scattering intensities in a coordinate system with the reciprocal-lattice vectors of the crystal as basis vectors (*i.e.* in coordinates of *h*, *k* and *l*). To do this, the orientation matrix is needed to convert between laboratory and crystal coordinates. This may be determined using implementations of standard algorithms (Kabsch, 1988*a* ▸, 1993 ▸) in custom scripts or alternatively using standalone programs with the algorithms already implemented (Guérin *et al.*, 2022 ▸; Kabsch, 2010 ▸).

During reconstruction of the diffuse scattering in reciprocal space, the data must be corrected for polarization (Kabsch, 1988*b* ▸). Furthermore, due to the use of a flat detector a solid-angle correction must also be applied. This correction scales the intensities of pixels on the detector with respect to the inverse of the solid angle each pixel subtends with respect to the sample. These two corrections are applied as a pixel-dependent scaling after performing the frame-by-frame subtraction of the averaged air scattering. After the corrections, the scattering intensities are mapped into the 3D reciprocal-space array. During this process, any pixels mapped into the same voxel of the 3D array are averaged. It is worth noting that, to ease the masking of Bragg peaks later in the data processing, it is helpful to choose a reciprocal-space grid where the Bragg peaks will always be centred in terms of voxel indices – this is for instance the case if the step size of the grid is 0.05 reciprocal-lattice units.

### 2.3. Symmetrization and outlier rejection

After reconstruction of the raw scattering data, we end up with a 3D scattering pattern, which partially fills reciprocal space (up to some scattering vector magnitude 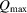). However, to Fourier transform the data, the data should be complete up to 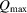. To achieve this, the data are usually symmetrized according to the point-group symmetry of the Laue class. Sometimes the diffuse scattering is of lower symmetry, for instance in the case of stacking disorder (Støckler *et al.*, 2022 ▸), and here care must be taken in this step. The symmetrization may be performed by applying the symmetry operations as matrix multiplications to the array indices (taking into account that the symmetry elements all go through the centre of the array). Each symmetry-equivalent voxel is then assigned the average values of the symmetry-equivalent voxels. During this symmetrization process it is beneficial to perform an outlier rejection, such as the one first used by Sangiorgio *et al.* (2018 ▸) inspired by the outlier rejection developed by Blessing (1997 ▸). In this case a voxel intensity, 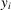, is considered an outlier if
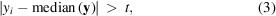
where **y** is a vector of all *n* measured symmetry-equivalent voxels and *t* is given by
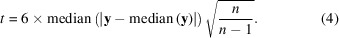

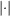
 here denotes the element-wise absolute value. Such an outlier rejection has proven immensely successful for removing *e.g.* small amounts of parasitic scattering which can otherwise prove problematic for 3D-ΔPDF analysis.

### 2.4. Punch and fill

The final step in the data treatment before Fourier transforming the diffuse scattering data to obtain the 3D-ΔPDF is to remove the Bragg peaks. This process is usually referred to as ‘punch and fill’ and may be done in a variety of ways as highlighted by Koch *et al.* (2021 ▸), with the simplest being applying an isotropic punch to the Bragg positions (clearing the values in a spherical region centred on the Bragg peak) and filling the missing intensities by linear interpolation. According to Koch *et al.* (2021 ▸), a suitable size for the punches may be chosen by first choosing a size significantly larger than the instrument resolution function and gradually decreasing it. The features of the 3D-ΔPDF will undergo significant changes when the punch size becomes too small as a consequence of Bragg diffraction intensity contributions. As such, the optimal size is the smallest size for which no abrupt changes to the 3D-ΔPDF are observed. However, if the diffuse scattering of interest is located far from the Bragg peaks it may be of interest to punch volumes extending beyond the Bragg diffraction intensities. This has to do with the fact that thermal diffuse scattering peaks are present underneath and in the immediate vicinity of the Bragg peaks (Willis & Pryor, 1975 ▸). By punching larger volumes, such as done by Simonov *et al.* (2020 ▸), this contribution is effectively removed, rendering the interpretation of the resulting 3D-ΔPDF more straightforward.

### 2.5. Fourier transform

The last step in the workflow to obtain the 3D-ΔPDF is Fourier transformation of the filled array. This can be done using the fast Fourier transform approximation implemented in standard libraries like Python’s *NumPy* (Harris *et al.*, 2020 ▸) and will thus not be covered in detail here. However, it is worth considering the resolution of the obtained 3D-ΔPDF. The discrete Fourier transform (DFT) on an interval 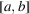 samples the Fourier coefficients such that the output array has the same size as the input array. The DFT samples points spaced with 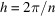, where *n* is the size of the array. If the sample frequency (the spacing of the points in the array) is 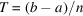, then 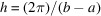 (Boggess & Narcowich, 2009 ▸). The length of the interval is 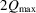. Thus, 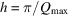. This means that the resolution of the 3D-ΔPDF is determined solely by the extent measured in reciprocal space.

## 3. Experimental

All in-house measurements were conducted on the Rigaku XtaLAB Synergy-S diffractometer at Aarhus University equipped with a HyPix-Arc 100° detector with Mo *K*α_1_ and *K*α_2_ radiation, and a sample-to-detector distance of 40 mm on the exact same crystals that were used for the synchrotron measurements. For all measurements, samples were glued to the end of glass pins using ep­oxy glue (Roth & Iversen, 2019 ▸; Roth *et al.*, 2021 ▸; Støckler, Zhang *et al.*, 2024 ▸). During the in-house measurements, these glass pins were mounted on a kappa-geometry goniometer.

All measurements on Cu_1.95_Se were conducted at 300 K on a crystal with dimensions of approximately 50 × 50 × 50 µm (Roth & Iversen, 2019 ▸). However, for the in-house measurements, an energy threshold of 14 keV was used (see the supporting information for a description of fluorescence effects). The corresponding Cu_1.95_Se synchrotron measurements were conducted on the 15-ID-D beamline on the APS using a photon energy of 40 keV and a Dectris Pilatus3 X 1M CdTe detector with a sample-to-detector distance of 120 mm. The sample glass pins were mounted on a Huber kappa-geometry goniometer (Roth & Iversen, 2019 ▸).

For the HH samples, all measurements were conducted at 300 K on crystals with dimensions of 30 × 30 × 40 µm and 40 × 25 × 10 µm for the slowly cooled and quenched samples, respectively. In-house data were collected for two of the samples used by Roth *et al.* (2021 ▸). The samples are labelled Q-0.84 #2 and SC-0.81 for quenched (Q) and slowly cooled (SC). The numbers refer to their nominal Nb content while the #2 suffix refers to the authors’ original sample number. However, as shown by Roth *et al.* (2021 ▸), the real stoichiometry is the same for the two samples (around *x* ∼ 1/6). The synchrotron measurements for the HH crystals were conducted on the BL02B1 beamline at SPring-8 with a Pilatus3 X 1M CdTe detector using a photon energy of 50.00 keV. The sample-to-detector distance was 130 mm for the quenched and 260 mm for the slowly cooled samples. The sample glass pins were mounted on a Huber four-circle (quarter-chi) goniometer (Roth *et al.*, 2021 ▸).

Finally, all InTe measurements were made at 100 K on a crystal with a size of approximately 100 × 50 × 30 µm. The synchrotron measurements were conducted at the BL02B1 beamline at the SPring-8 synchrotron equipped with a Pilatus3 X 1M CdTe detector with a photon energy of 47.00 keV and an energy threshold of 34.00 keV. The sample glass pins were mounted on a Huber four-circle (quarter-chi) goniometer (Støckler, Zhang *et al.*, 2024 ▸).

All the diffuse scattering measurements described above were followed by air scattering measurements for background subtraction. For these measurements, the sample pin was retracted from the beam. All parameters were kept the same for the air scattering except for the angular range of the frame, which was increased (see the supporting information).

During the reconstruction, background subtraction was applied along with polarization and solid-angle corrections. The grid sizes for Cu_1.95_Se and Nb_1−*x*_CoSb for the synchrotron measurements were 901 × 901 × 910 voxels (Roth & Iversen, 2019 ▸; Roth *et al.*, 2021 ▸), while they were 401 × 401 × 501 voxels and 601 × 601 × 601 voxels for the in-house Cu_1.95_Se and Nb_1−*x*_ CoSb, respectively. For InTe, the grid sizes were 601 × 601 × 601 voxels for the synchrotron measurements and 649 × 649 × 649 voxels for the in-house measurements. During symmetrization, the point-group symmetry of the Laue class of the crystal was applied to the diffuse scattering.

As for the punch and fill step, all punched voxels were filled by linear interpolation, but the punch shapes differed between the measurements. Cylindrical rods parallel to the c* axis with a diameter of 5 voxels were used for the in-house Cu_1.95_Se data while spheres with a diameter of 15 voxels were used for the Nb_1−*x*_CoSb and InTe in-house data. Outside the data regions on the in-house measurement, the non-measured voxels were filled with random Gaussian noise to resemble the background. For the synchrotron measurements on Nb_1−*x*_CoSb, a 3D spline interpolation was used with spherical punches and the regions outside the measured data were filled with a constant value (Roth *et al.*, 2021 ▸). Spherical punches with a diameter of 9 voxels were used for synchrotron InTe data. The punched areas were filled with a linear interpolation while the areas outside the measured data were filled with Gaussian noise. For the in-house data on InTe, the punches were likewise spherical, but with a diameter of 15 voxels. Here, the regions outside the measured data were also filled with Gaussian noise to resemble the background.

## 4. Results and discussion

### 4.1. Disorder in Cu_1.95_Se

**Figure 1:**
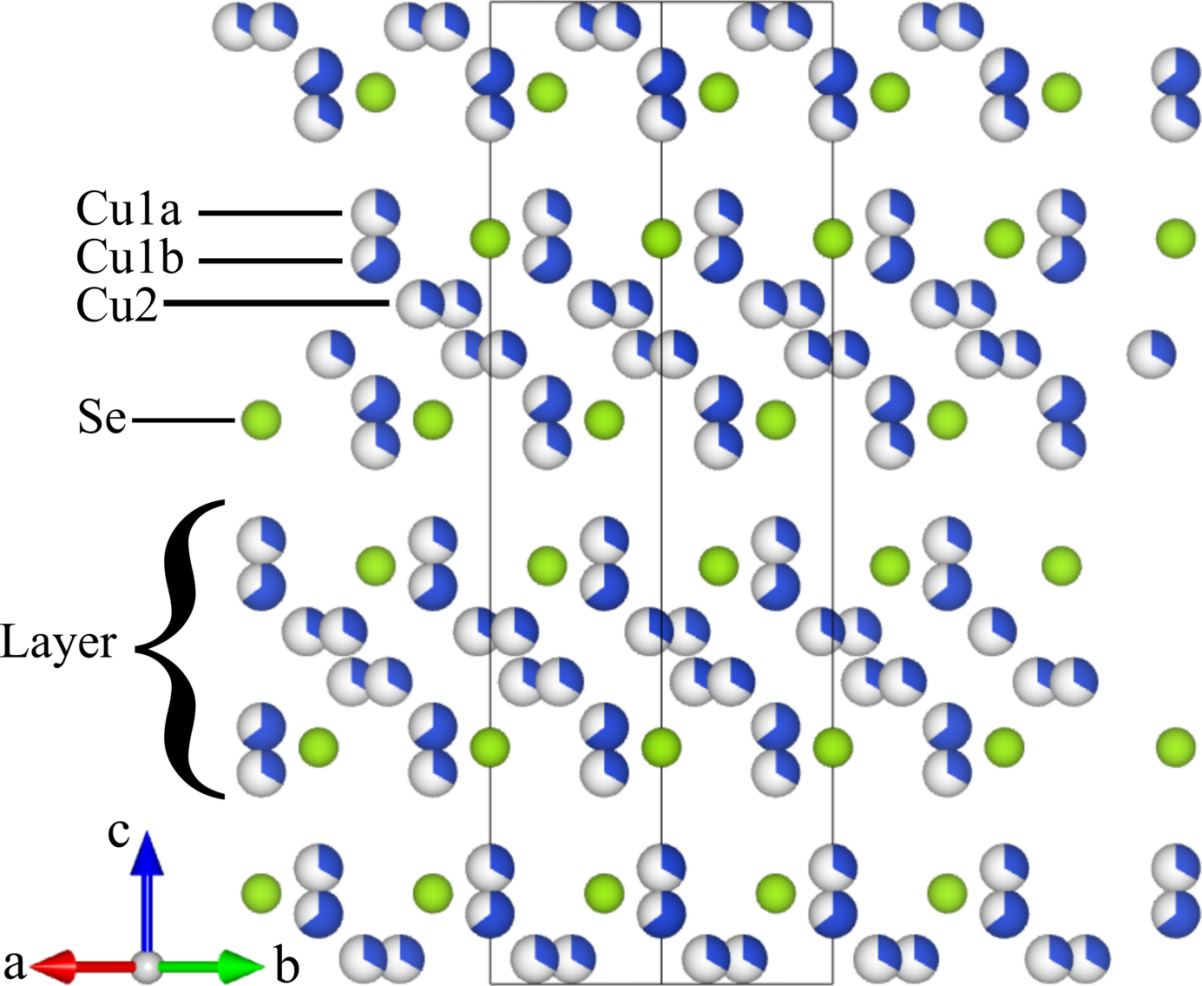
The average unit cell of Cu_1.95_Se found by Roth & Iversen (2019 ▸). Cu atoms are blue while Se atoms are green. Partial occupancy is marked by filling fractions. Atoms are arranged in layers perpendicular to the *c* axis (marked ‘Layer’). Based on analysis of the diffuse scattering these layers are completely ordered in the real structure (see text). Each layer consists of sublayers marked Cu1a, Cu1b, Cu2 and Se (two of each sublayer are present in each layer) which are also perpendicular to the *c* axis. The figure was made with *VESTA* (Momma & Izumi, 2011 ▸).

The average structure of Cu_1.95_Se was determined by Eikeland *et al.* (2017 ▸) and the real structure containing correlated disorder was subsequently solved by Roth & Iversen (2019 ▸). Eikeland *et al.* (2017 ▸) found the structure to belong to space group 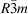 with unit-cell axes of 4.1217 (3) Å and 20.435 (3) Å for *a* and *c*, respectively (see Table 1 ▸). The average unit cell is shown in Fig. 1 ▸. As can be seen, it consists of layers stacked along the *c* axis. Each layer consists of sublayers of Cu or Se.

**Figure 2:**
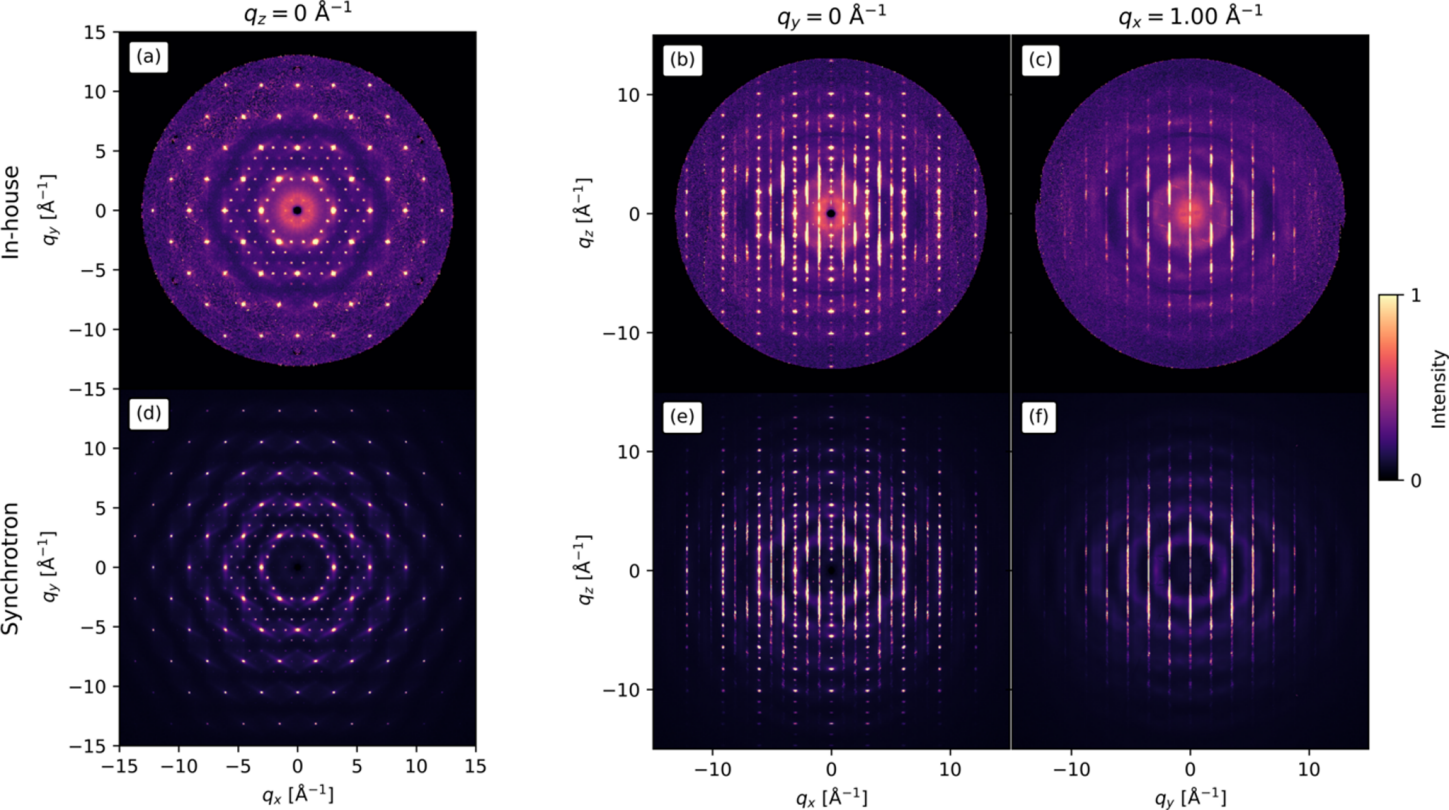
The scattering patterns of Cu_1.95_Se from the (*a*)–(*c*) in-house and (*d*)–(*f*) synchrotron measurements. (*a*) and (*d*) are in the 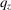 = 0 Å^−1^ plane while (*b*) and (*e*) are in the 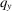 = 0 Å^−1^ plane, and (*c*) and (*f*) are in the 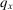 = 1.00 Å^−1^ plane. The scattering patterns are very similar except for larger 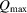 and less noise in the synchrotron scattering patterns. Intensity in each row is on an arbitrary scale.

The scattering patterns from the in-house and synchrotron measurements are shown in Fig. 2 ▸. The intense spots are Bragg peaks while the lines and smaller spots are diffuse scattering. To any visible extent, the scattering patterns appear similar although the signal-to-background ratio is less for the in-house measurements compared with the synchrotron measurements. As can be seen, the diffuse scattering does not overlap with the Bragg peaks. This is fortunate, since there will be no danger of punching the diffuse scattering along with the Bragg peaks during the data reduction. Due to the use of both Mo *K*α_1_ and *K*α_2_ in the in-house measurements, the peaks have a radial broadening. This is clearest from the Bragg peaks in Fig. 2 ▸(*a*).

**Figure 3:**
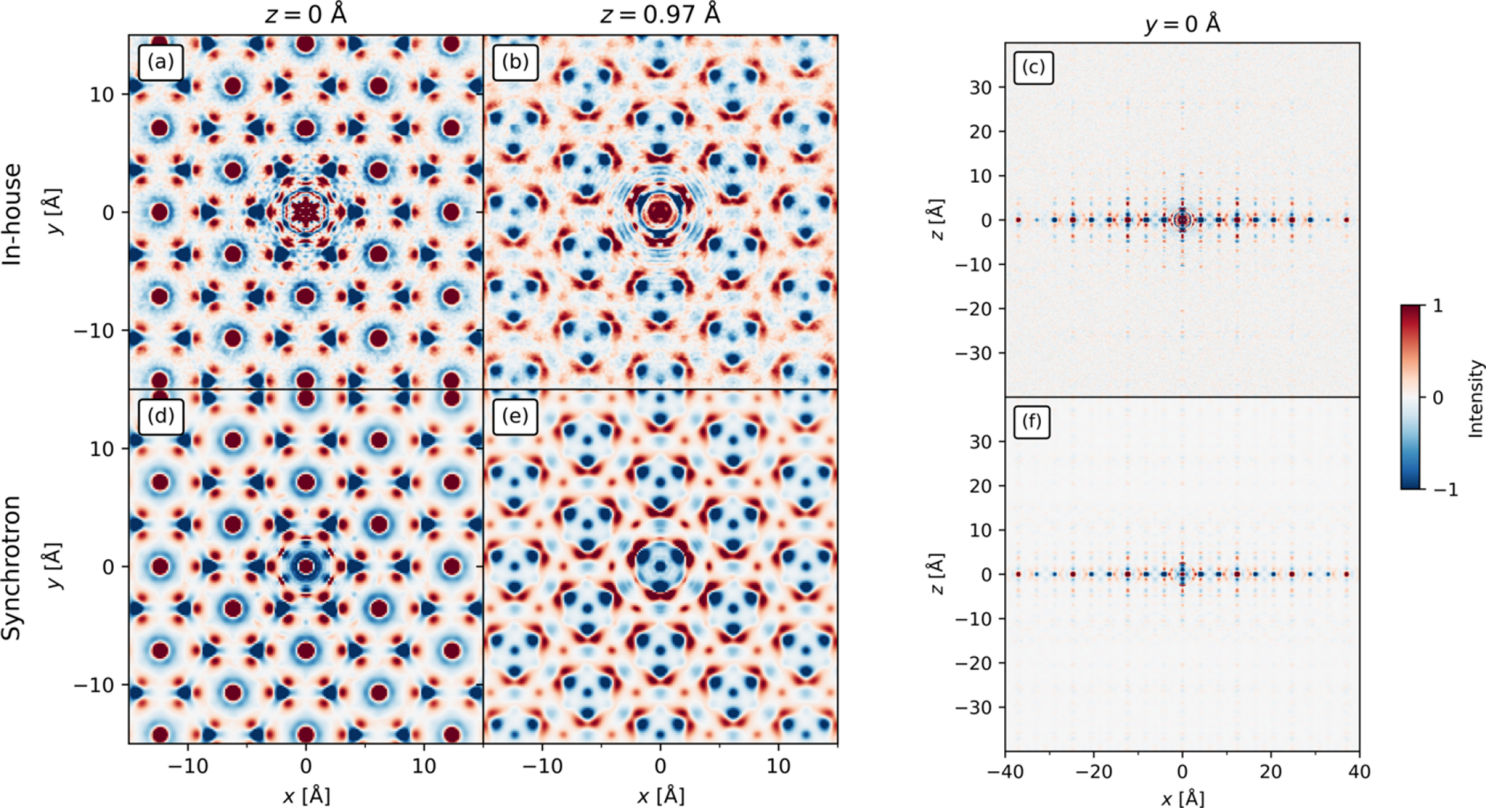
The 3D-ΔPDFs of Cu_1.95_Se from the (*a*)–(*c*) in-house and (*d*)–(*f*) synchrotron measurements in the (*a*) and (*d*) *z* = 0 Å plane, (*b*) and (*e*) the *z* = 0.97 Å plane and (*c*) and (*f*) *y* = 0 Å plane. The 3D-ΔPDFs are visually identical except for noise near the origin of the in-house 3D-ΔPDF which changes a few of the analysis steps, but it is not detrimental to the analysis (see text). The intensity in each row is on an arbitrary scale.

The radial broadening in the in-house measurements is carried over to the 3D-ΔPDF (Weber & Simonov, 2012 ▸). This is visible for the negative rings surrounding the intense positive peaks in Fig. 3 ▸(*a*) as they are more intense towards the origin of the 3D-ΔPDF (the Mexican hat features). However, this does not affect the qualitative analysis of the 3D-ΔPDF.

Another note of interest is the extent to which the data were measured in *Q* space as this determines the resolution of the 3D-ΔPDF. For the in-house measurements, 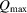 = 13 Å^−1^ which is seen as the extent of the data in Fig. 2 ▸. For the synchrotron measurements, 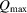 = 25.76 Å^−1^ (not shown). While the in-house diffractometer can go to a higher 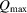, this requires a much longer measuring time. Using *Q* = 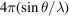

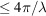
, one obtains a maximal *Q* value for Mo of 17.7 Å^−1^ (with a wavelength of 0.709 Å). Thus, a synchrotron is clearly favoured for obtaining a high 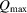.

It is worth noting that, apart from the noise near the origin and a rougher background, the 3D-ΔPDFs (see Fig. 3 ▸) from the in-house and synchrotron measurements contain the same features. Thus, only features close to the origin have been obscured. However, as will be shown below, this is not detrimental to the analysis of the in-house 3D-ΔPDF. In the comparison of the in-house 3D-ΔPDF with the synchrotron 3D-ΔPDF, it will be shown that it is possible to reach the same conclusions as Roth & Iversen (2019 ▸) found using the synchrotron 3D-ΔPDF.

**Figure 4:**
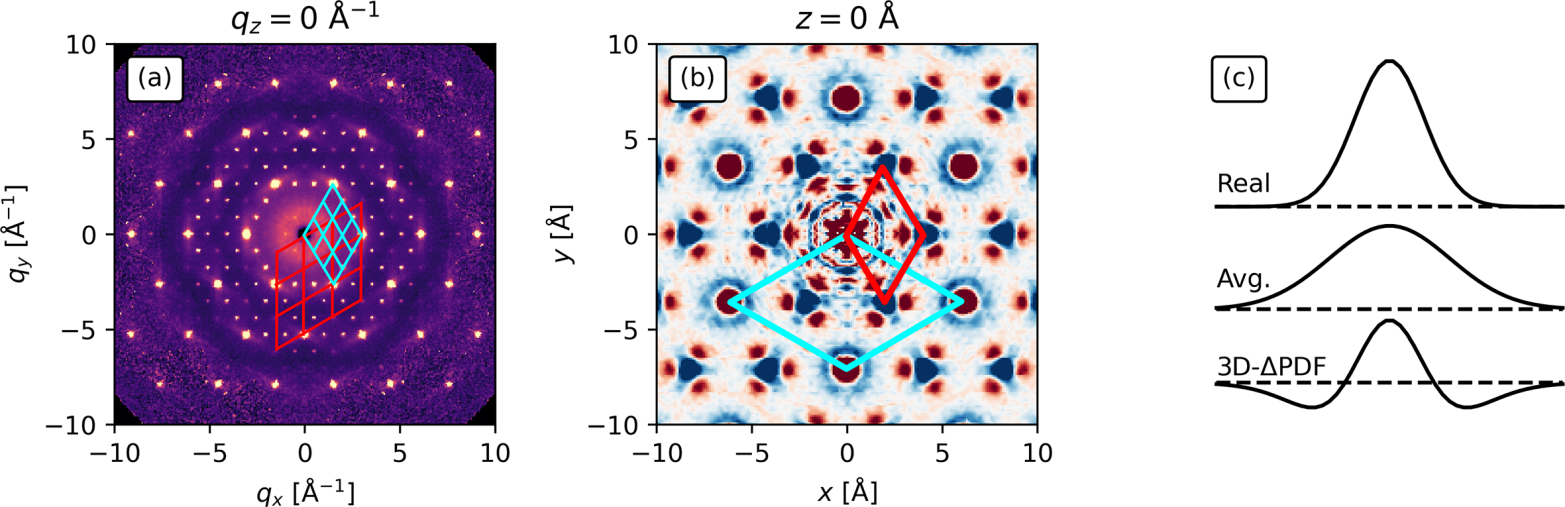
(*a*), (*b*) The average structure/unit cell for Cu_1.95_Se in the 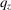 = 0 Å^−1^/*z* = 0 Å plane is shown in red while the supercell in the 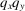 /*ab* plane is shown in blue. The average cell does not match with equivalent positions in the 3D-ΔPDF in (*b*) while the supercell does as the origin point must correspond to the other corners of the cell due to the inherent properties of the 3D-ΔPDF. (*c*) A schematic showing the 3D-PDF (top), the average structure Patterson function (middle) and the 3D-ΔPDF at the origin (the difference between the other two) (bottom).

The first thing to consider is that the total scattering in Fig. 2 ▸ shows rods of diffuse scattering parallel to the 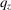 axis at non-integer *h* and *k* values. The non-integer *h* and *k* nature of the rods can be rationalized from Fig. 4 ▸(*a*) where the red cells show part of the reciprocal lattice of the average structure. The rods are then seen as the weaker spots within each cell not indexed by the basis vectors of the cell. Rods of diffuse scattering are indicative of loss of periodic order in 1D (along the direction parallel to the rods) while sharp Bragg peaks along the remaining axes indicate that complete order is preserved along those directions. Thus, it describes a 1D disorder (and thus 2D order) commonly referred to as stacking disorder. Such disorder is characterized by completely ordered layers stacked in a correlated, but disordered fashion (Sears *et al.*, 2023 ▸; Støckler *et al.*, 2022 ▸). This suggests an ordered superstructure in the *ab* plane [also seen in Fig. 4 ▸(*a*)]. As the environment around each lattice point must be identical (by definition of the lattice), all corners of the unit cell must have identical features in the 3D-ΔPDF (be at equivalent positions). Looking at Fig. 4 ▸(*b*), the vectors corresponding to the unit-cell *a* and *b* axes do not correspond to equivalent positions in the 3D-ΔPDF (highlighted in red) as the origin has a different feature compared with the other corners. Instead, the supercell can be constructed as shown in Fig. 4 ▸(*b*) (highlighted in cyan). This new supercell also describes the smaller spots observed in Fig. 4 ▸(*a*), which are in fact rods of diffuse scattering (as described in Fig. 2 ▸). The observation of the 2D supercell eases the analysis of the layers in the corresponding plane. This supercell was also found by Roth & Iversen (2019 ▸).

While the origin of the in-house 3D-ΔPDF is obscured by noise (in contrast to the synchrotron 3D-ΔPDF), the origin feature must always be a positive circle surrounded by a negative ring. This can be rationalized using the schematic in Fig. 4 ▸(*c*) and remembering that the 3D-ΔPDF feature at the origin corresponds to the self-correlation of atoms. The 3D-PDF shown in the top of Fig. 4 ▸(*c*) is a narrow peak, since it corresponds to the autocorrelation of the atomic electron density with itself. On the other hand, the average structure Patterson function in the middle row of Fig. 4 ▸(*c*) is the autocorrelation of the average, thermally smeared atomic electron density with itself and is therefore broader. Since the 3D-ΔPDF is the difference between these two, it will consist of a positive peak surrounded by negative values, as illustrated in the bottom row of Fig. 4 ▸(*c*).

**Figure 5:**
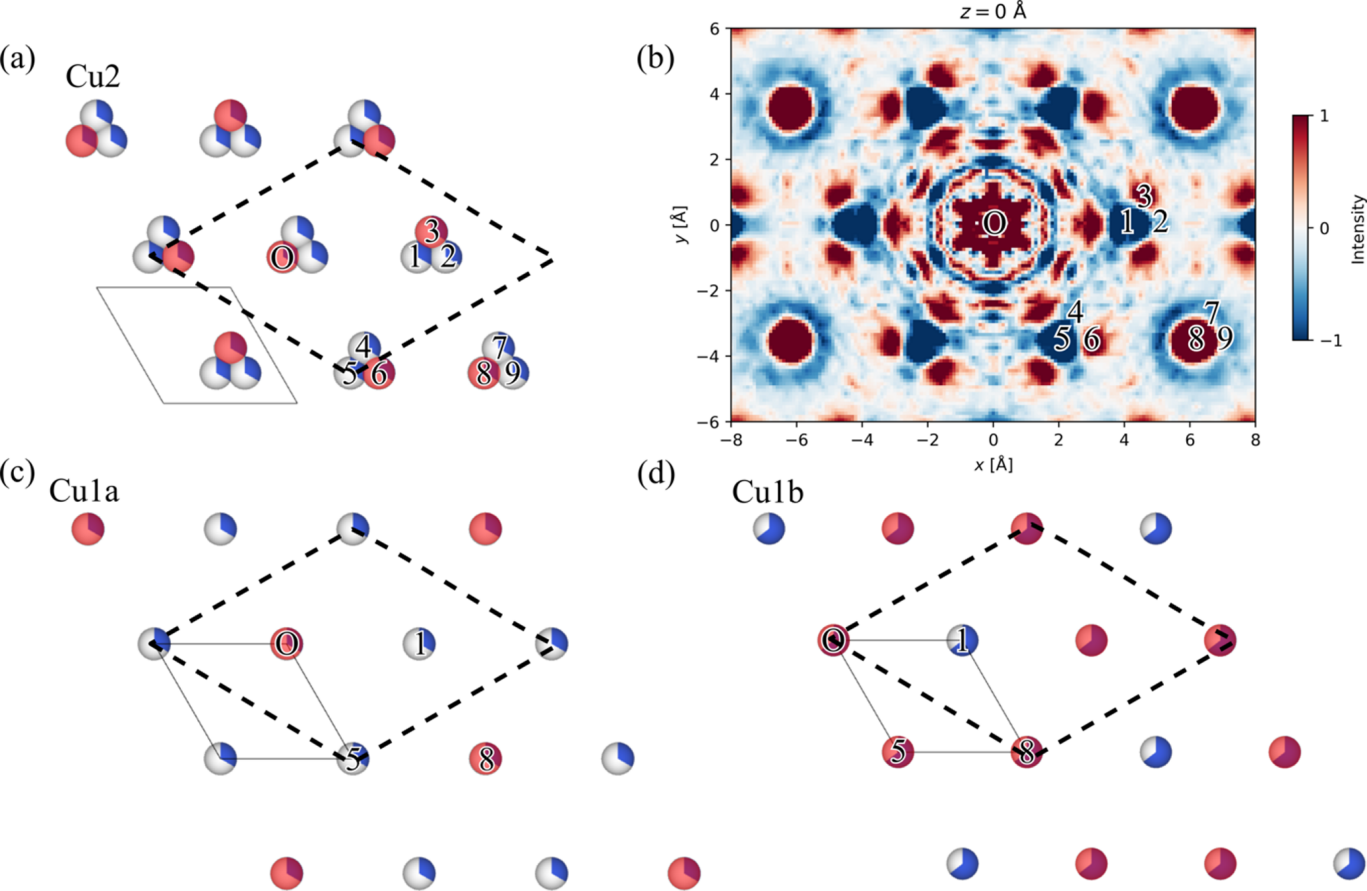
A single layer of Cu atoms (seen in the *ab* plane) of the (*a*) Cu2 sublayer, (*c*) Cu1a sublayer and (*d*) Cu1b sublayer. Red-tinted atoms mark the occupied sites while the rest are unoccupied. The dashed areas mark the ordered unit cell (supercell) while the fully drawn areas mark the average unit cell. The numbers on the atoms correspond to the location of the atom relative to the origin ‘O’ in the (*b*) 3D-ΔPDF. The intensity is on an arbitrary scale.

Firstly, the Cu sublayers perpendicular to the *c* axis are analysed (see Fig. 5 ▸). This begins by choosing the origin of each layer (‘O’) to be occupied. For the Cu2 layer [Fig. 5 ▸(*a*)], Roth & Iversen (2019 ▸) noted that only one atom per cluster is occupied by observing that the origin peak in the 3D-ΔPDF [Fig. 3 ▸(*d*)] is surrounded by a negative ring. This feature is obscured in the in-house 3D-ΔPDF [Fig. 3 ▸(*a*)]. However, this can be bypassed in one of two ways. The first is based on the knowledge that the ionic radius of Cu(I) is between 0.60 and 0.79 Å, depending on environment (Weller *et al.*, 2018 ▸). Having Cu(I) ions 0.88 Å apart, which is significantly less than twice their ionic radii, is chemically unreasonable. The other way to see this is from the 3D-ΔPDF utilizing the 2D order of the layers. Another origin (like site ‘8’) could have been chosen. As this is also surrounded by a negative ring, the same conclusion can be reached.

To assign occupied sites of the individual sublayers, one notes that the ordered cell in the Cu1a and Cu1b sublayers contains three atomic sites. With the sites of the individual sublayers having an occupancy of 1/3 and 2/3, respectively, the number of atoms in each sublayer within the ordered cell must be one and two, respectively. The Cu2 layer contains nine atomic sites per supercell, each with an occupancy of 1/3, giving a total of three Cu atoms per supercell within this sublayer. Occupied sites in the Cu2 and Cu1a sublayers can then be assigned through the atomic sites corresponding to positive correlations in the 3D-ΔPDF, as shown in Figs. 5 ▸(*a*) and 5 ▸(*c*). A more thorough description of how to manually analyse this 3D-ΔPDF is given in the original publication by Roth & Iversen (2019 ▸).

In the Cu1b layer [Fig. 5 ▸(*d*)], the atoms at sites ‘1’ and ‘8’ can be assigned as unoccupied and occupied, respectively, due to their respective negative/positive features in the 3D-ΔPDF. The site at ‘5’ needs some more work. Using equation (2), Roth & Iversen (2019 ▸) showed that this site needs to be occupied. Using the same argument, the occupancy at ‘5’ can be shown from the O1 interatomic vector. By counting the appearance of this and symmetry-equivalent vectors, it can be found that 1/3 of these vectors separate two occupied sites and 2/3 of these vectors separate an occupied site from an unoccupied site. This gives an integral proportional to 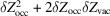 = [(1/3)^2^ + 2 × (1/3) × (−2/3)]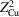 = 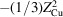. Thus, it should have a negative integral. This intra-sublayer analysis is all in agreement with the findings of Roth & Iversen (2019 ▸).

**Figure 6:**
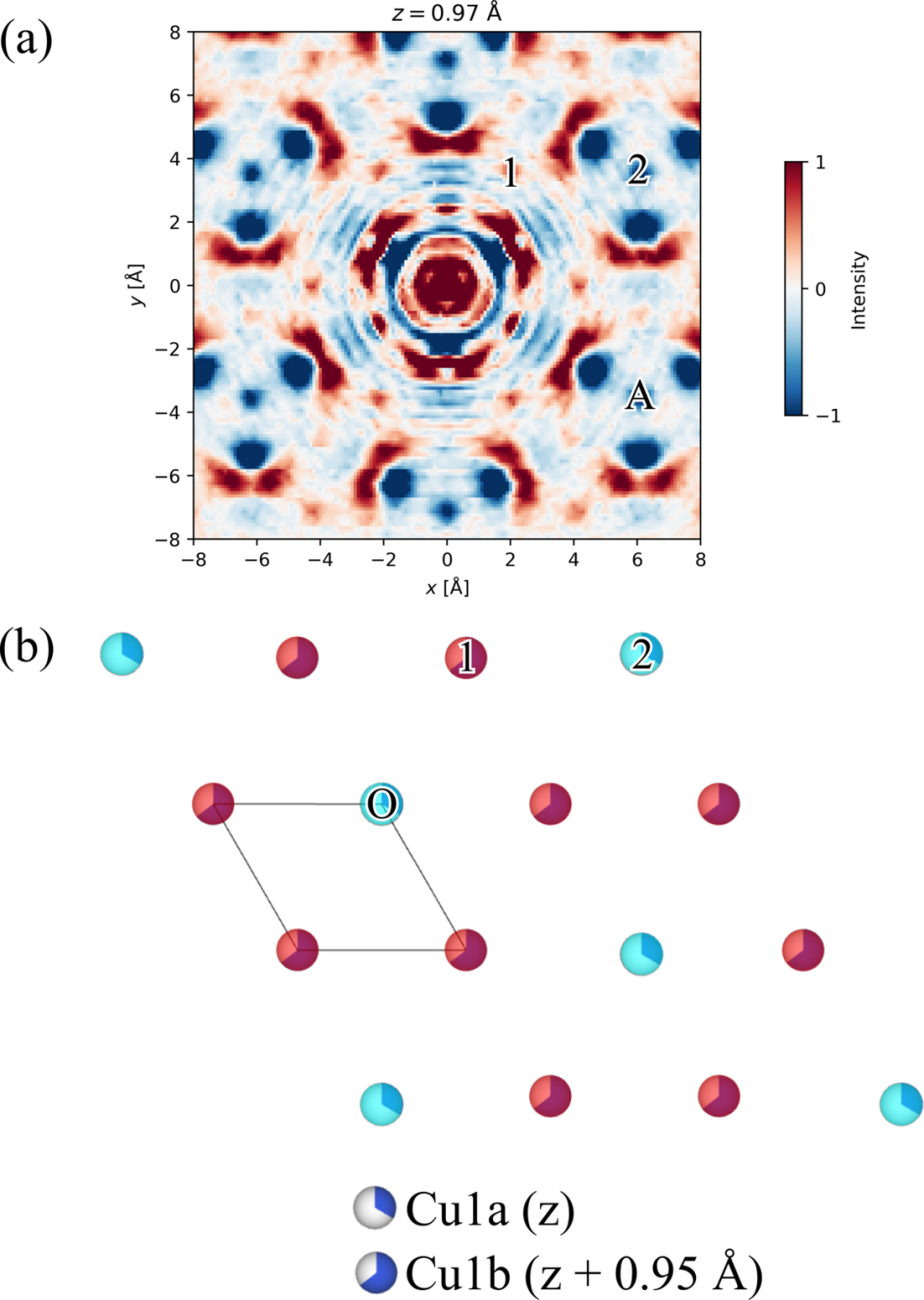
(*a*) The *z* = 0.97 Å layer in the 3D-ΔPDF which is the slice closest to the *z* component of the inter-sublayer orderings between the Cu1a and Cu1b sublayers in Cu_1.95_Se. (*b*) The inter-sublayer order of the Cu1a and Cu1b sublayers. The blue-toned atoms mark the occupied sites in the lower sublayer while the red-tinted atoms mark the occupied sites in the upper sublayer. The numbers on the atoms correspond to the location of the atom relative to the origin of the 3D-ΔPDF. The intensity is on an arbitrary scale.

For the inter-sublayer order, Roth & Iversen (2019 ▸) found the inter-sublayer distance for the Cu1a and Cu1b layers to be 0.95 Å. The closest grid points to this are at *z* = 0.97 Å in both the synchrotron and in-house 3D-ΔPDFs. The other inter-sublayer correlations can also be analysed from that plane (Fig. 6 ▸).

Looking at the vector (0, 0, 0.97 Å), which is at the origin of Figs. 3 ▸(*b*) and 3 ▸(*e*), Roth & Iversen (2019 ▸) found that only a single site can be occupied in each Cu1a–Cu1b dimer at a time due to the negative peak at this location in the synchrotron 3D-ΔPDF. This feature is obscured by noise in the in-house 3D-ΔPDF. However, this can be bypassed by observing the peak marked ‘8’ in Fig. 5 ▸(*b*) and assuming it is occupied in the Cu1b layer. The feature marked ‘A’ in Fig. 6 ▸(*a*) thus corresponds to the atom marked ‘5’ in the Cu1a layer in Fig. 5 ▸(*c*). The negative feature at ‘A’ in Fig. 6 ▸(*a*) leads to the same conclusion as that of Roth & Iversen (2019 ▸), which was that the Cu1a site must be unoccupied if the Cu1b atom is present. It is possible to utilize the origin shift due to the 2D order in the layers. If this had been absent, it might not have been possible to assign this inter-sublayer feature. Alternatively, ambiguity can be resolved by again considering the ionic radius of Cu(I) – an interatomic distance of 0.95 Å is not chemically sensible. Only one inter-sublayer arrangement is possible, if the requirement of neighbouring sites of the Cu1a and Cu1b sublayers to not be simultaneously occupied is to be fulfilled for all Cu1a–Cu1b pairs [Fig. 6 ▸(*b*)]. This is further supported by the features marked ‘1’ and ‘2’ in Fig. 6 ▸(*a*). The feature at ‘1’ is weak in the in-house 3D-ΔPDF, but as it is larger than the noise and matches an interatomic vector, it should be considered. Analysis of the other layers can be found in the supporting information.

The stacking disorder is equally visible in the in-house and synchrotron 3D-ΔPDFs [Figs. 3 ▸(*c*) and 3 ▸(*f*)] as the correlations extend far in the *x* direction while decaying fast in the *z* direction. The same features are visible in the in-house and synchrotron 3D-ΔPDFs and they extend equally far in direct space. Overall, the most important difference between the in-house and synchrotron 3D-ΔPDFs is the more prominent noise in the in-house 3D-ΔPDF. However, as has been shown, it was not prohibitive for the analysis. Either arguments based on the 2D order or on ionic radii can be utilized to solve the correlated disorder using the in-house 3D-ΔPDF. This may not be possible for other systems, and in such cases synchrotron experiments may be required. To better examine the effects of the noise, a system with weaker diffuse scattering and no 2D order will be examined.

### 4.2. Nb_1−*x*_CoSb half-Heuslers

**Figure 7:**
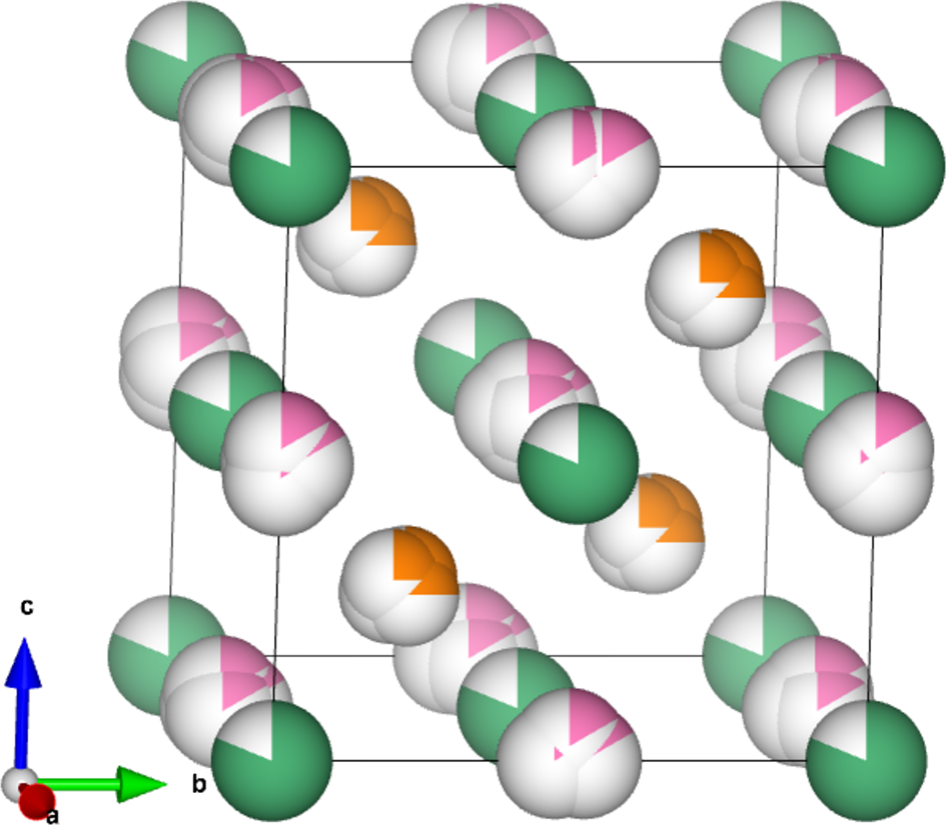
The average unit cell found by Roth *et al.* (2021 ▸) for both HH compounds. Nb (green) has a fractional occupancy 0.82 for the SC-0.81 crystal and 0.83 for the Q-0.84 #2 crystal. Co (orange) is split into four sites with 1/4 fractional occupancy each, and Sb (pink) is split into six sites each with a fractional occupancy of 1/6 (Roth *et al.*, 2021 ▸). The figure was made with *VESTA* (Momma & Izumi, 2011 ▸).

In the original synchrotron study of defects in HH compounds, Roth *et al.* (2021 ▸) found the disorder to be size effects. This is displacive disorder stimulated by substitutional disorder (Weber & Simonov, 2012 ▸). Size effects visible from the average unit cell are shown in Fig. 7 ▸. In their analysis Roth *et al.* (2021 ▸) integrated the peaks of the 3D-ΔPDF in order to decouple the displacive and substitutional disorder. In this study, the sensitivity of the integrals to box size will be investigated as it is expected that the larger noise in the in-house 3D-ΔPDF will affect the integrals.

**Figure 8:**
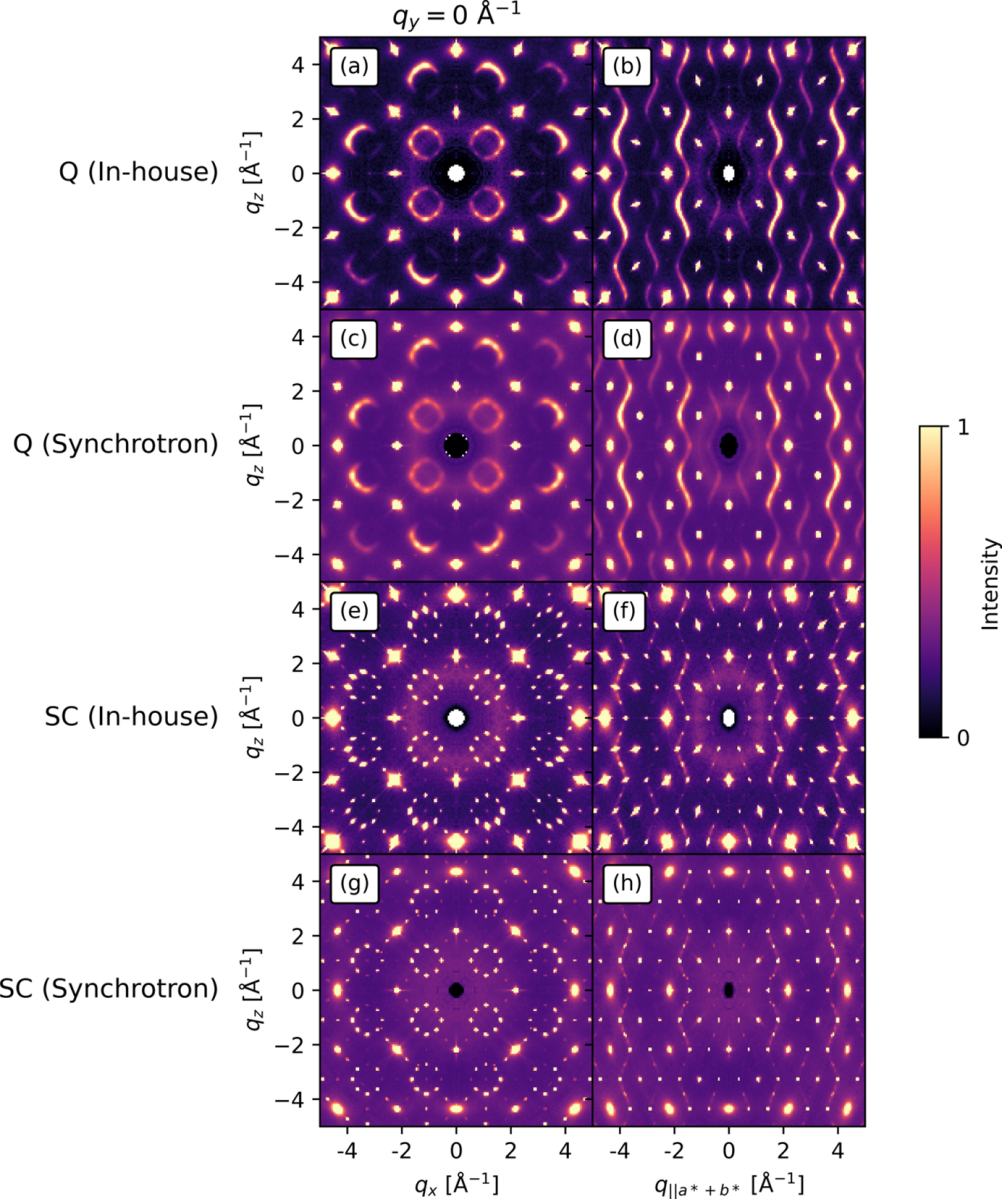
The scattering patterns for the (*a*), (*b*) quenched (Q) sample in-house and (*c*), (*d*) quenched sample from the synchrotron, (*e*), (*f*) slowly cooled (SC) sample in-house, and (*g*), (*h*) slowly cooled sample from the synchrotron. The scattering patterns are shown in the 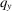 = 0 Å^−1^ plane, corresponding to the *h*0*l* plane (left column), and the plane perpendicular to 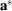
–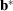, corresponding to the *hhl* plane (right column). The increased background on the synchrotron measurements is due to the lack of fluorescence suppression on these measurements. The intensity in each row is on an arbitrary scale. A discussion on the effect of the difference in resolution can be found in the supporting information.

In-house and synchrotron single-crystal X-ray scattering data for the quenched and slowly cooled crystals are shown in Fig. 8 ▸ in the 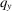 = 0 Å^−1^ plane and the plane perpendicular to 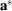
–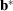 (*i.e.* the *h*0*l* and *hhl* planes). The Bragg peaks in the in-house measurements [Figs. 8 ▸(*a*), 8 ▸(*b*) and 8 ▸(*e*), 8 ▸(*f*)] have a radial broadening, which is absent from the corresponding synchrotron measurements [Figs. 8 ▸(*c*), 8 ▸(*d*) and 8 ▸(*g*), 8 ▸(*h*)]. The in-house measurements have a lower background, which is due to applied energy discrimination on the detector. The rings of the diffuse scattering in the 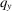 = 0 Å^−1^ plane (Fig. 8 ▸, left column) have higher intensity away from the origin, which Roth *et al.* (2021 ▸) found to be due to size effects. For the slowly cooled sample [Figs. 8 ▸(*e*), 8 ▸(*h*)], the rings condense into peaks in the 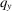 = 0 Å^−1^ plane which suggests that there is a longer-range order in that structure. Similar to Cu_1.95_Se, the Bragg peaks still do not overlap with the diffuse scattering, so punching the Bragg peaks is not problematic for these samples.

**Figure 9:**
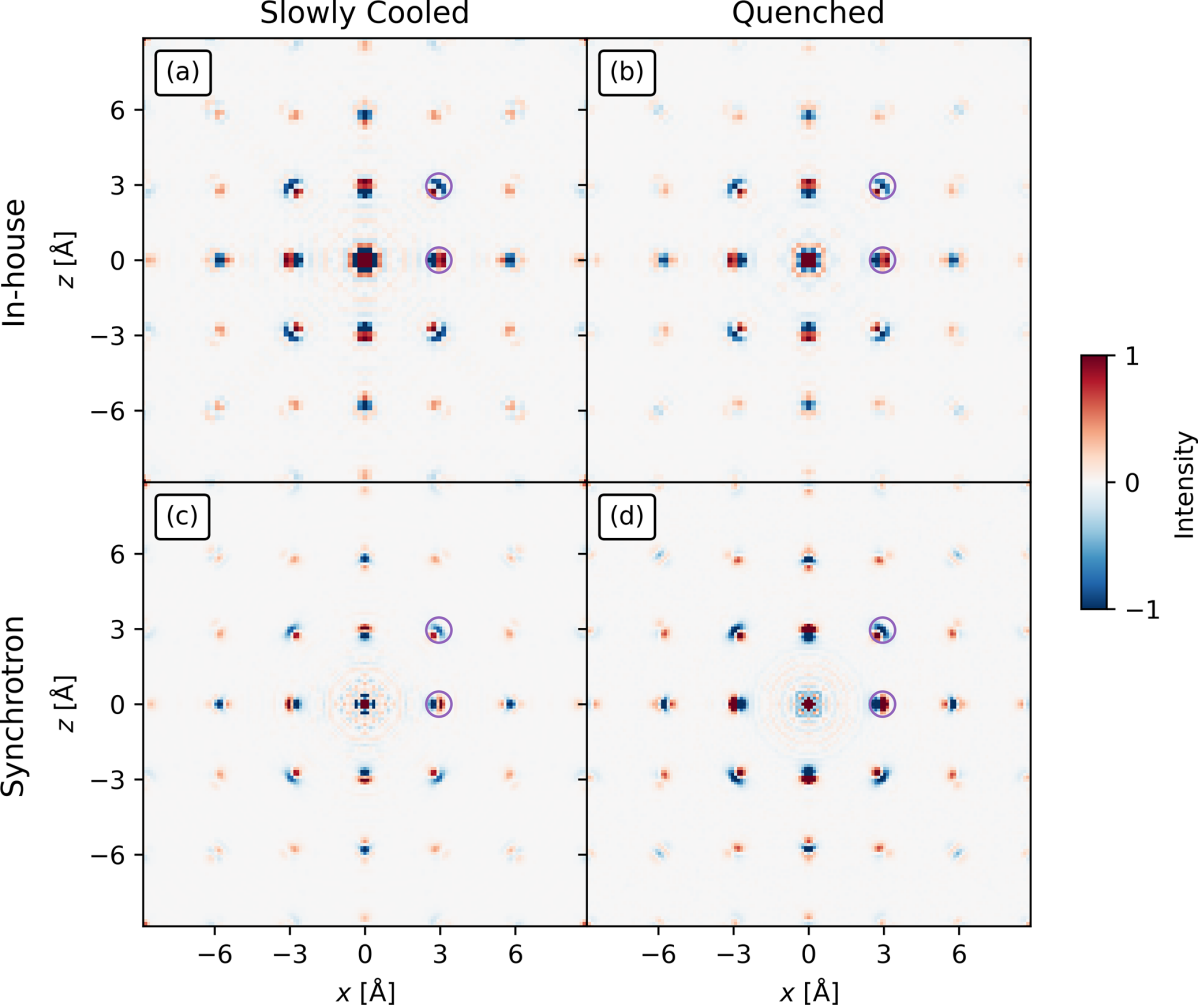
The 3D-ΔPDFs from the HH measurements on (*a*), (*b*) in-house and (*c*), (*d*) synchrotron. (*a*) and (*c*) are from the slowly cooled sample and (*b*) and (*d*) are from the quenched sample. The noise level is of the same order of magnitude for the in-house and synchrotron measurements, but the resolution is still better on the synchrotron measurements. Intensity in each panel is on an arbitrary scale. The features used during the analysis in the main text are marked with purple rings. A discussion on the effect of the difference in resolution can be found in the supporting information.

When comparing 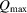 between the measurements, the synchrotron measurements were conducted up to 25.76 Å^−1^ while the in-house measurements were conducted to 16 Å^−1^; this gives synchrotron 3D-ΔPDFs better resolution than the in-house, as seen in Fig. 9 ▸, yet the same features are visible in the in-house 3D-ΔPDF. In contrast to the Cu_1.95_Se sample, the in-house and synchrotron 3D-ΔPDFs have noise in the same order of magnitude, which does not challenge the analysis.

The feature at (2.95 Å, 0, 0) in the 3D-ΔPDFs in Figs. 9 ▸(*a*), 9 ▸(*b*) corresponds to the Sb–Nb vector or the Sb-vacancy vector. The negative feature on the side closer to the origin and positive away from it suggests that Sb will move further away when a Nb atom is present at the origin. In contrast, the feature at (2.95 Å, 0, 2.95 Å) which corresponds to the Nb–Nb interatomic vector suggests that the Nb atoms prefer to move closer together if both are present and away from a vacancy. These conclusions are identical to those made by Roth *et al.* (2021 ▸) based on the synchrotron 3D-ΔPDF, but now made with only the in-house 3D-ΔPDF.

This type of feature (size effects) is composed of two components: substitutional disorder and displacive disorder. It is interesting to be able to decouple these effects and analyse them individually. The peak integral is given by equation (2). As the integral relates only to differences in the number of electrons separated by a particular vector, it is affected only by substitutional disorder. As such, it is possible to isolate the effect of the substitutional disorder by analysing only the integrated intensities of each peak (Roth *et al.*, 2021 ▸; Roth & Iversen, 2019 ▸). The integration method used by Roth *et al.* (2021 ▸) is box integration which will therefore also be used here.

**Figure 10:**
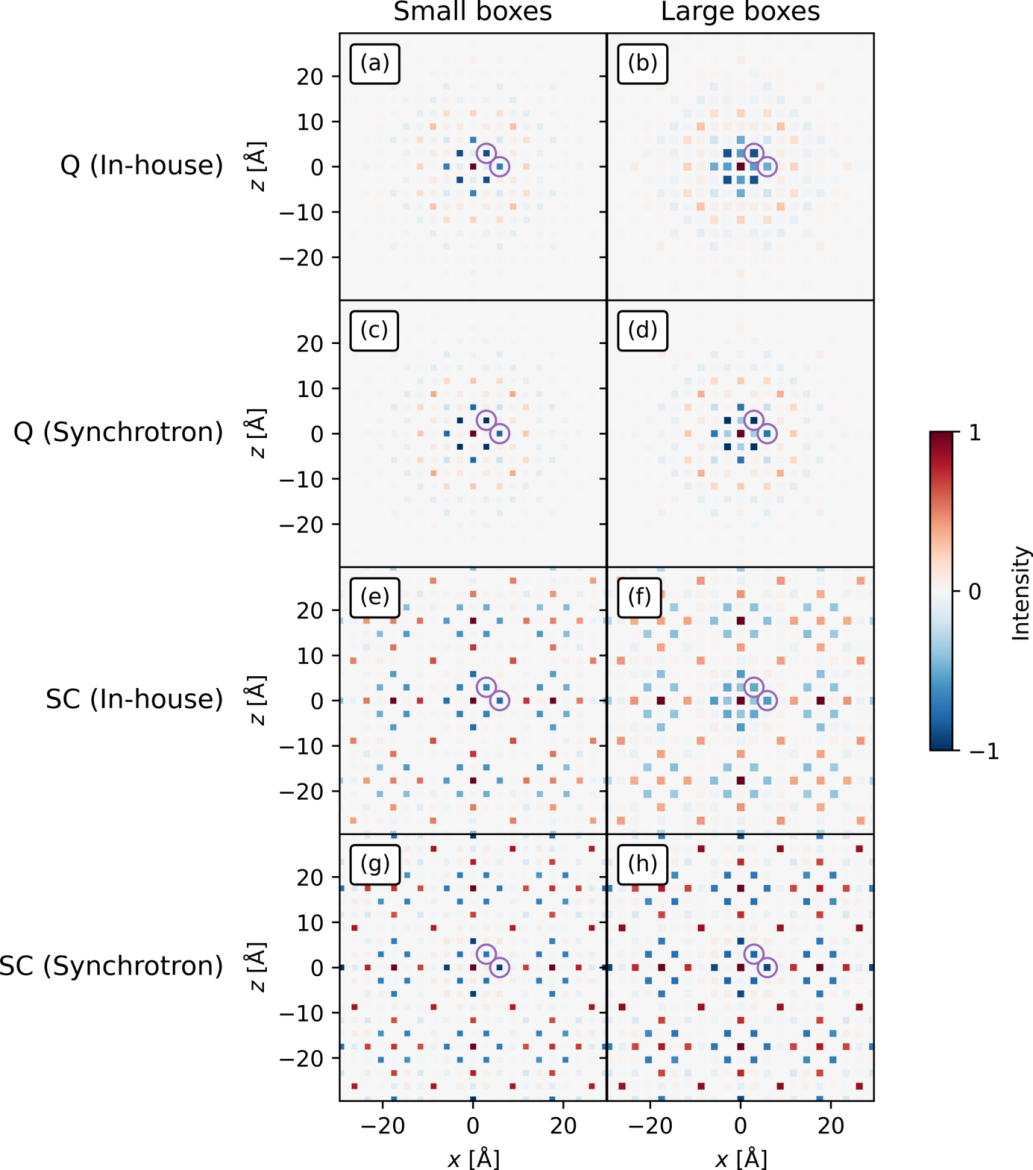
Integrated peaks from the (*a*), (*b*) quenched in-house, (*c*), (*d*) quenched synchrotron, (*e*), (*f*) slowly cooled in-house, and (*g*), (*h*) slowly cooled synchrotron samples. The left column shows small boxes which are 7 × 7 × 7 voxels and 11 × 11 × 11 voxels for the in-house and synchrotron measurements, respectively. The right column shows large boxes which are 9 × 9 × 9 voxels and 13 × 13 × 13 voxels for the in-house and synchrotron measurements, respectively. The greatest difference is near the origin in the in-house measurements where the noise is greatest. The small boxes for the synchrotron data were used by Roth *et al.* (2021 ▸) in their analysis. The intensity in each row is on an arbitrary scale. The features used in the main text are marked with purple rings.

As the in-house measurements contain asymmetric radial broadening from the use of both Mo *K*α_1_ and *K*α_2_ radiation, the effect of the choice of box size is expected to be more pronounced than for synchrotron measurements, where monochromatic radiation limits the broadening of the 3D-ΔPDF features (see the supporting information). This effect will therefore be examined using box integration as this is the method used by Roth *et al.* (2021 ▸). A box should contain the entire feature and thus it should be larger than the extent of said feature. Using larger boxes than the smallest encapsulating box would start to include background into the integral, which is undesirable. The radially broadened reflections in the in-house data suggest that larger box sizes would be needed which leads to the danger of including more background in the integration. To test the effect of larger box sizes, two different box sizes were constructed. The box sizes used for the in-house 3D-ΔPDF were 7 × 7 × 7 voxels and 9 × 9 × 9 voxels. For the synchrotron 3D-ΔPDF, the box sizes were 11 × 11 × 11 voxels and 13 × 13 × 13 voxels. In both cases, the smallest boxes were the boxes that visually exactly covered the main peak. The integration boxes were made centred on 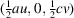 where 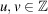, and *a*, *c* are the lengths of the unit-cell axes. The procedure is described in the supporting information. The real-space extent of the smallest boxes was 1.34 Å on the synchrotron versus 1.37 Å on in-house. The results are shown in Fig. 10 ▸. As the figure shows, the different integration box sizes only affect the integrals of the peaks closest to the origin to any significant extent. Since these vectors separate Nb and Sb, the integral should vanish since 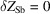. Thus, the non-zero integral obtained with larger box sizes must be attributed to noise. The in-house integrals are affected more by larger boxes due to the relatively larger noise close to the origin [Figs. 10 ▸(*b*), 10 ▸(*f*)]. However, as the synchrotron quenched sample shows, the integrals from the synchrotron data are also affected by the box size [Fig. 10 ▸(*d*)], although the effect is negligible for the slowly cooled sample [Fig. 10 ▸(*h*)]. Thus, it is important to choose proper integration boxes for the synchrotron measurements, but more so for the in-house data. This would be the boxes that fully accommodate the peaks while still resulting in vanishing integrals for vectors where at least one of 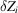 or 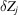 is equal to zero. Furthermore, our analysis shows that the radial broadening on in-house measurements did not require the use of larger box sizes.

The integrals from the in-house data can be analysed equivalently to how Roth *et al.* (2021 ▸) interpreted the synchrotron integrals for the small boxes. Looking at the integrals at (2.95 Å, 0, 2.95 Å) and (5.9 Å, 0, 0), the negative features represent a preference for avoiding nearest- and next-nearest-neighbour vacancies. Thus, they must correspond to vacancy-Nb vectors. The positive features at longer distances represent the preferred vacancy–vacancy distances. This is seen by placing a vacancy at the origin and using equation (2) which gives an integral proportional to 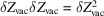 = 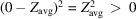 for the vacancy–vacancy peaks. These features are all equally visible from the in-house and synchrotron data. Thus, the same conclusions can be reached using either data set, separately.

It has now been demonstrated that the in-house method can be used on systems with weaker diffuse scattering which does not overlap with the Bragg peaks. The radial broadening of the peaks did not change the integrals significantly. However, in the case of overlap between the diffuse scattering and the Bragg peaks, the radial broadening of the peaks in the in-house method may become problematic for the separation of Bragg and diffuse scattering.

### 4.3. InTe

In the thermoelectric material InTe, vacancies within the 1D In^+^ chains induce Frenkel defects (Zhang *et al.*, 2021 ▸). The local structure associated with the formation of these defects was solved based on single-crystal diffuse scattering from Pb-doped InTe (nominal stoichiometry Pb_0.01_In_0.99_Te) and pure InTe by Støckler, Zhang *et al.* (2024 ▸) using the 3D-ΔPDF method. However, the data used for solving this local structure were collected at 25 K, which better allowed discernment of important features in the 3D-ΔPDF.

As such low temperatures cannot be obtained using standard nitro­gen cryostreamers on in-house instruments, 100 K synchrotron and in-house data are used here to make a better comparison. However, it should be noted that going to lower temperatures is an advantage, as it allows additional temperature effects to be removed and the conditions for modelling the real structure to be improved. The focus of this section will thus be visual inspection of the 3D-ΔPDF, and the features used by Støckler, Zhang *et al.* (2024 ▸) to solve the local defect structure which are also present in the synchrotron 3D-ΔPDF at 100 K.

**Figure 11:**
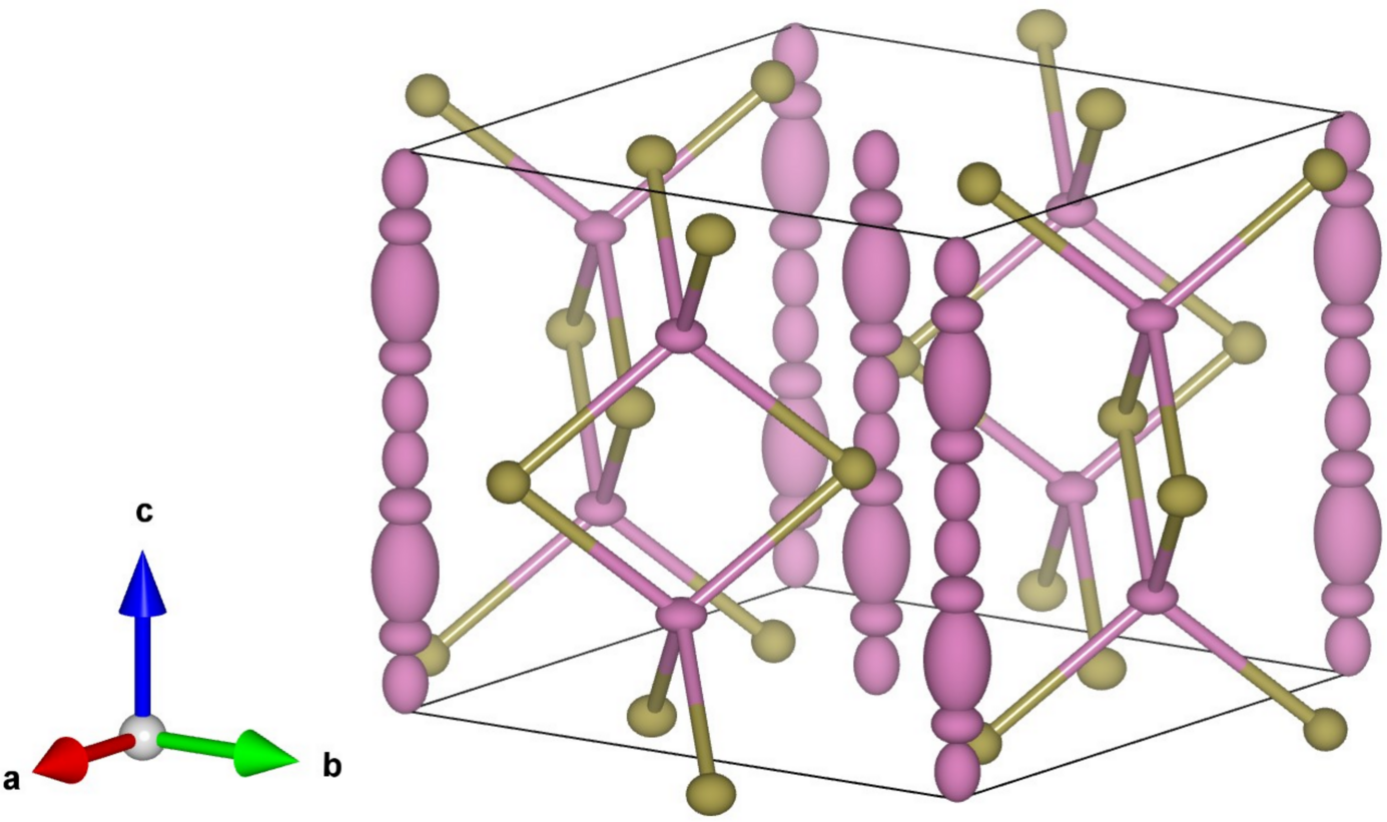
The 100 K average structure of pure InTe refined to In_0.982_Te (Støckler, Zhang *et al.*, 2024 ▸). The magenta atoms are In while the gold-coloured ones are Te. The chains parallel to the *c* axis are In^+^ ions with the main site being the site with the large *U*_33_ component. The other sites are interstitial sites with much lower occupancy than the main site (not shown). The figure was made with *VESTA* (Momma & Izumi, 2011 ▸).

The average structure of InTe is shown in Fig. 11 ▸ where the disordered chains of In^+^ ions oriented along the *c* axis are clearly observed. The disorder in the chains is a result of vacancy-induced Frenkel defects in the structure, where the In^+^ ion on the main site (large displacement ellipsoid) is displaced into interstitial sites along the chain. The large *U*_33_ component of the main In^+^ site also indicates displacive disorder along the *c* axis due to structural relaxations near the vacancy defects. Between the chains are tetrahedra of In^3+^Te_4_^2−^. The tetrahedrally coordinated In^3+^ ions have oblate displacement ellipsoids, showing greatest displacements perpendicular to the *c* axis and towards the neighbouring In^+^ chains. As for the Te^2−^, the prolate displacement ellipsoids have their main axis towards the nearest In^+^ chains. This is due to displacive disorder coupling to the substitutional disorder in the chains (Støckler, Zhang *et al.*, 2024 ▸).

**Figure 12:**
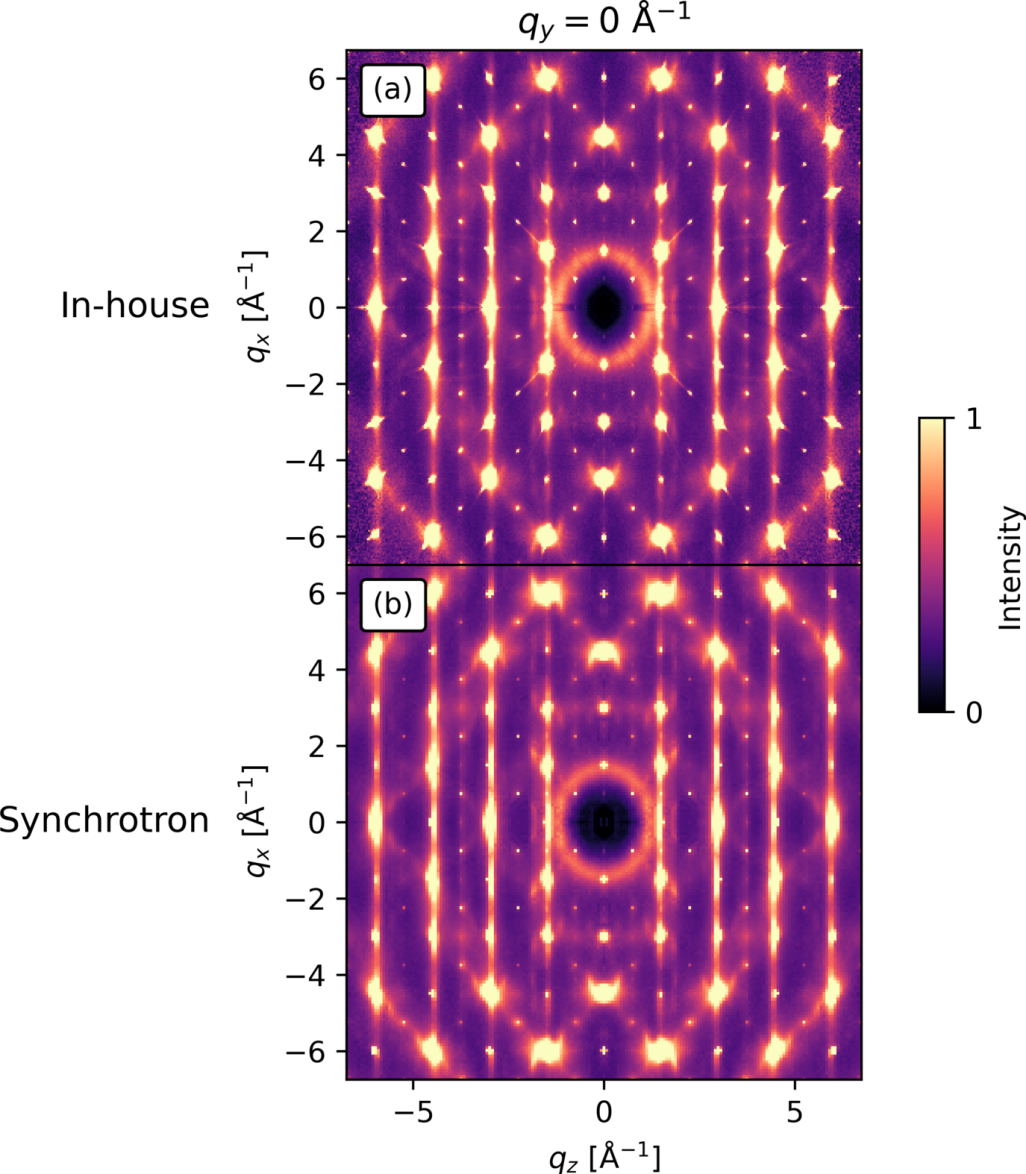
Total scattering of InTe from (*a*) in-house and (*b*) synchrotron experiments both shown in the 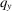 = 0 Å^−1^ plane (*h*0*l* plane). In both (*a*) and (*b*) planes of diffuse scattering overlapping with the Bragg peaks are observed along with the radial broadening in the in-house measurements (*a*) which was also observed for the other samples. The intensity in each row is on an arbitrary scale.

The diffuse scattering consists of planes, which are observed in Fig. 12 ▸ as lines perpendicular to the 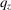 axis. The main challenge here, compared with the previous examples, is that the diffuse scattering planes overlap with the Bragg peaks. Once again, radial broadening is observed for the in-house sample, which leads to larger volumes punched during data reduction. Apart from this, the only visible difference between the in-house and synchrotron measurements is the 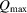. The 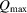 with full completeness is 12.4 Å^−1^ and 12.8 Å^−1^ with partial completeness in the in-house data compared with 15.2 Å^−1^ full completeness and 22.5 Å^−1^ partial completeness in the synchrotron data (not shown).

**Figure 13:**
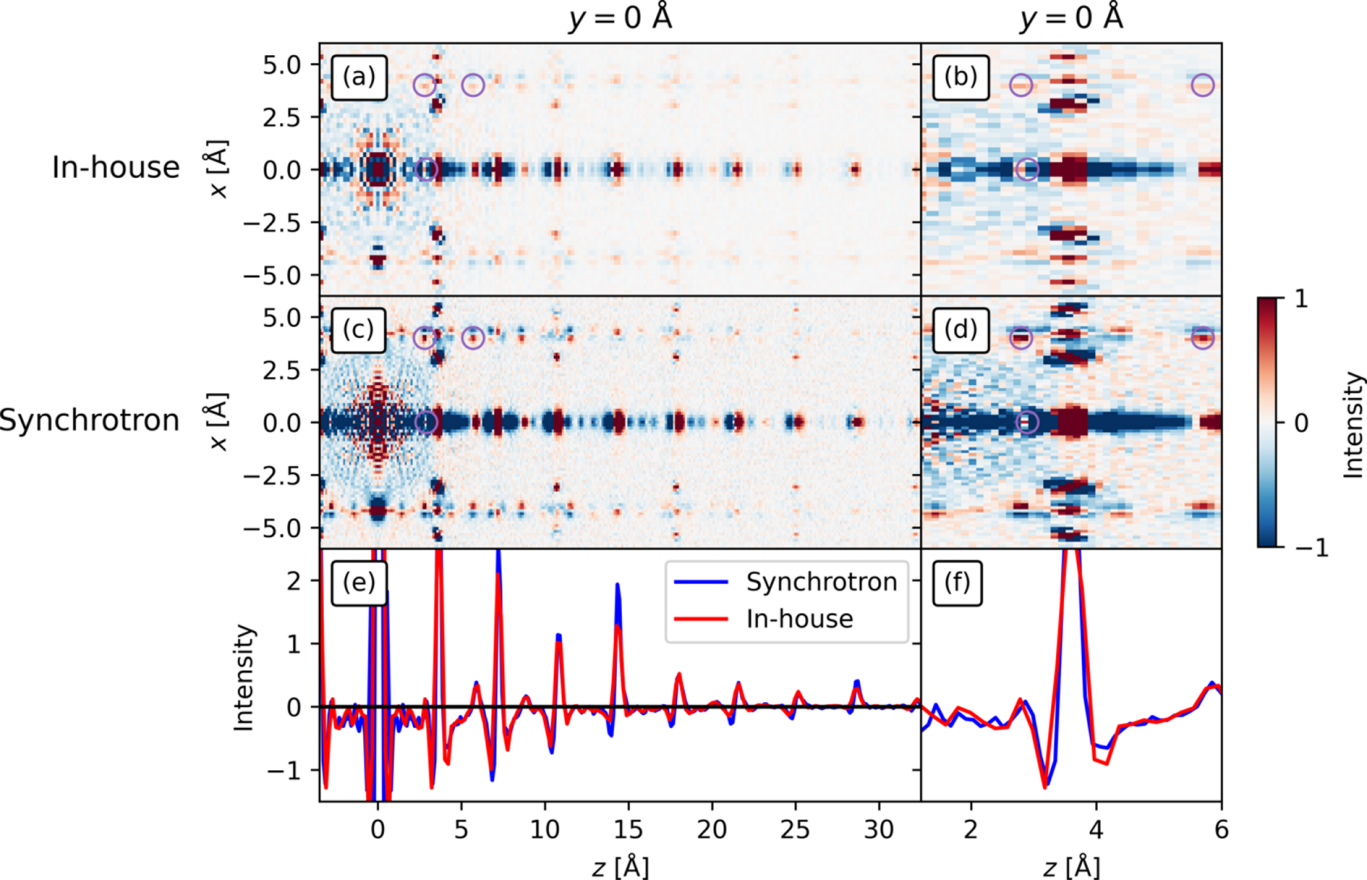
The 3D-ΔPDFs for (*a*) in-house and (*c*) synchrotron measurements on InTe in the *x*0*z* plane (*x* is parallel to *a* and *z* is parallel to *c*). (*e*) The profile of the 3D-ΔPDFs for the voxels in the (0, 0, *z*) line. All figures are on different scales, including the two profiles in (*e*). The intensity is on an arbitrary scale. (*b*), (*d*) and (*f*) are close-ups of the discussed features near the origin.

Concerning the 3D-ΔPDFs shown in Fig. 13 ▸, most of the important features for the model are visible in both 3D-ΔPDFs [Figs. 13 ▸(*a*) and 13 ▸(*c*)]. In the (0, 0, *z*) line, some of the longer-range features are more clearly visible above the noise in the synchrotron 3D-ΔPDF, as was also the case for the previous examples. Starting from low *z* in the (0, 0, *z*) line, the synchrotron 3D-ΔPDF is negative below *z* = 2.8 Å after the noise region, which Støckler, Zhang *et al.* (2024 ▸) interpreted as being due to such short interatomic distances being less likely than in the average structure. This is also seen in the in-house 3D-ΔPDF, although the noise and lower resolution make the negative feature less visible. Even so, it is still above the noise level in the later part of the region, similar to the synchrotron 3D-ΔPDF, which also has noise in the low-*z* part of that region.

The feature located approximately at (0, 0, 3.0 Å) was assigned by Støckler, Zhang *et al.* (2024 ▸), using the synchrotron 3D-ΔPDF. This feature is also visible in the in-house 3D-ΔPDF even though it is only a single voxel in size. It should also be considered as a feature due to it being in the (0, 0, *z*) direction where the signal is much above the noise level. Støckler, Zhang *et al.* (2024 ▸) assigned this as a correlation between occupied interstitial sites in the In^+^ chain. Assigning this feature correctly to the correlation between two interstitial sites and not to a correlation between a main and interstitial site in the chain requires the analysis of the size effect where In^3+^ relaxes towards or away from the In^+^ chain. The size effect observed in the 3D-ΔPDF as features located at (4 Å, 0, 2.6 Å) and (4 Å, 0, 5.6 Å) is practically indistinguishable from the noise in the in-house 3D-ΔPDF. Here, we see the first example where the lower resolution and lower signal-to-noise ratio in the in-house data lead to obscured features. Additionally, Fig. 13 ▸(*c*) shows the features along the (0, 0, *z*) line to be more radially broadened in the in-house 3D-ΔPDF compared with the synchrotron 3D-ΔPDF, as expected. This also obscures the features from the in-house 3D-ΔPDF. Thus, assigning the (0, 0, 3.0 Å) feature correctly may be difficult with the in-house 3D-ΔPDF. This assignment played an important role in solving the puzzle of the disorder surrounding the In^+^ vacancies, since it describes the ordering of interstitials within the Frenkel defect region of the structure. As such, it is improbable that one would be able to solve the local structure of InTe based on the in-house 3D-ΔPDF shown here. We summarize the discussion with a table of the most important comparisons (see Table 2 ▸).

### 4.4. Relations to quantum crystallography

In studies of correlated disorder, the typical approach is to develop and refine models of the disorder phenomena possibly based on ‘interaction’ parameters. However, such interaction models are phenomenological, whereas the fundamental origin of correlated disorder is some specific local chemical bonding preferences that must be fulfilled. Thus, a more complete description of correlated disorder phenomena would require scattering models that consider the chemical nature of the atoms in the crystals. So far, all models of correlated disorder have used independent atom model scattering factors, which lack chemical information apart from the atomic identity. In the presence of disorder, the local chemical environments of the atoms can be very different even for sites equivalent in the average structure unit cell. For example, in the case of LuFe_2_O_4_, the mixed-valence Fe^2+^/Fe^3+^ sites have very different coordination environments depending on the specific iron-atom valence (Støckler, Roth *et al.*, 2024 ▸). Similarly, the correlated disorder in bixbyite, Fe_2−*x*_Mn_*x*_O_3_, is driven by local Jahn–Teller effects of the metal atoms (Støckler *et al.*, 2022 ▸). Methodologies to introduce precalculated scattering factors based on aspherical and charged atomic electron densities in the modelling of local structures presumably will improve the fits to the observed diffuse scattering data if they correctly reflect the local chemical bonding. In modelling of average structure from Bragg intensities, refinement with predetermined aspherical scattering factors was proposed by Lecomte and coworkers (Pichon-Pesme *et al.*, 1995 ▸), and later a range of aspherical scattering factor databanks were introduced to increase the accuracy of crystal structure modelling (Dittrich *et al.*, 2006 ▸; Volkov *et al.*, 2004 ▸). More recently, ‘on the fly’ theoretical calculation of aspherical Hirshfeld atom scattering factors has been used in refinement of (organic) molecular crystal structures (Jayatilaka & Dittrich, 2008 ▸). The models using such fixed (*i.e.* non-refined) aspherical scattering factors improve the positions of hydrogen atoms (the most ‘severe’ aspherical atoms) in average crystal structure refinement, but as shown by Dominiak and coworkers they are also useful in refinement of noisy low-resolution Bragg diffraction data (Jha *et al.*, 2023 ▸). Introduction of explicitly parametrized aspherical scattering factor models such as the multipole model (Stewart, 1976 ▸; Hirshfeld, 1977 ▸; Hansen & Coppens, 1978 ▸), refinable against diffuse scattering data, could ultimately allow the experimental data to expose the chemical origin of the disorder. However, this prospect is still somewhat distant.

## 5. Conclusion

Although examples from the literature show that in-house 3D-ΔPDF analysis is possible and has useful applications in local structure analysis of disordered crystals, no comparative study existed to relate the quality of in-house 3D-ΔPDFs to their synchrotron counterparts. By studying the exact same crystal specimens of Cu_1.95_Se, Nb_1−*x*_CoSb (HH) and InTe on both in-house and synchrotron sources, we have obtained directly comparable 3D-ΔPDFs. In the study of Cu_1.95_Se, the same 2D order was visible and the unique assignment of the atoms in the layers was possible. The main problem encountered during the in-house study was the lower signal-to-noise ratio, which meant that additional arguments based on the 2D order or ionic radii had to be utilized to solve the structure. For the HH crystals, it was possible to isolate the substitutional disorder and assign the same vacancy ordering as observed in the synchrotron analysis. The larger noise meant that the integration box size had to be chosen more carefully, but a method for ensuring a proper choice of box size was proposed. The radial broadening of the peaks in the in-house 3D-ΔPDF did not change the outcome of the integration. For InTe it was not possible to construct the same model as from the synchrotron data since the weak diffuse scattering, the radial broadening due to non-monochromatic radiation and the higher measurement temperature did not allow for the observation of the necessary features for constructing the full disorder model. Overall, we conclude that the in-house method is effective and useful for studies of correlated disorder, but, as with many other aspects of crystallography, the higher data resolution and better signal-to-noise ratio of synchrotron data lead to a more accurate and detailed crystal structure analysis.

## Supplementary Material

Supporting information. DOI: 10.1107/S2053273325003389/pl5047sup1.pdf

## Figures and Tables

**Table 1:** The unit-cell parameters for the different samples The parameters for Cu_1.95_Se are from Roth & Iversen (2019 ▸). The others were determined from the in-house data using *CrysAlisPRO* (Agilent, 2010 ▸). The two Nb_1−*x*_CoSb samples are named as Q/SC for quenched and slowly cooled, respectively, followed by their nominal Nb content. [For Q-0.84 #2, the final #2 refers to the authors’ sample numbering as used by Roth *et al.* (2021 ▸).]

Compound	Space group	*a* (Å)	*b* (Å)	*c* (Å)	α (°)	β (°)	γ (°)
Cu_1.95_Se	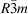	4.1217 (3)	4.1217 (3)	20.435 (3)	90	90	120
Nb_1−*x*_CoSb	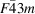						
Q-0.84 #2		5.9000 (7)	5.9000 (7)	5.9000 (7)	90	90	90
SC-0.81		5.8950 (6)	5.8950 (6)	5.8950 (6)	90	90	90
InTe	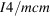	8.38261 (6)	8.38261 (6)	7.11880 (13)	90	90	90

**Table 2:** Some of the most important differences between synchrotron and in-house sources The comparison is general in terms of what is generally considered available/reasonable at the time of writing and is by no means complete or valid for all instruments.

	Synchrotron	In-house
Accessibility	Less accessible and travel costs	Very accessible, but with acquisition and maintenance costs
Radial broadening due to lack of monochromaticity	No	Yes
Beam divergence	Very small	Yes
Resolution	Very good	Medium (acceptable)
Signal-to-noise ratio	Very high/high	Medium/low
Duration of measurement	Hours (very high flux)	Hours/days (medium/low flux)
Temperature	Can often measure at very low temperatures	Can typically measure at moderately low temperatures
Difference between elemental sources	–	Higher atomic number gives better resolution, but lower scattering intensities (longer measurements)
Fluorescence?	Change wavelength and/or use fluorescence suppression on detector	Change source if possible and/or use fluorescence suppression on detector

## Data Availability

Data and code are available from the corresponding author upon reasonable request.

## References

[bb1] Agilent (2010). *CrysAlisPRO.* Agilent Technologies Ltd, Yarnton, Oxfordshire, England.

[bb2] Blessing, R. H. (1997). *J. Appl. Cryst.* **30**, 421–426.

[bb3] Boggess, A. & Narcowich, F. J. (2009). *A First Course in Wavelets with Fourier Analysis*, 2nd ed. John Wiley & Sons.

[bb4] Davenport, M. A., Krogstad, M. J., Whitt, L. M., Hu, C., Douglas, T. C., Ni, N., Rosenkranz, S., Osborn, R. & Allred, J. M. (2021). *Phys. Rev. Lett.* **127**, 125501. 10.1103/PhysRevLett.127.125501 34597061

[bb5] Dittrich, B., Strumpel, M., Schäfer, M., Spackman, M. A. & Koritsánszky, T. (2006). *Acta Cryst.* A**62**, 217–223. 10.1107/S0108767306010336 16614494

[bb6] Ehrling, S., Reynolds, E. M., Bon, V., Senkovska, I., Gorelik, T. E., Evans, J. D., Rauche, M., Mendt, M., Weiss, M. S., Pöppl, A., Brunner, E., Kaiser, U., Goodwin, A. L. & Kaskel, S. (2021). *Nat. Chem.* **13**, 568–574. 10.1038/s41557-021-00684-4 34045713

[bb7] Eikeland, E., Blichfeld, A. B., Borup, K. A., Zhao, K., Overgaard, J., Shi, X., Chen, L. & Iversen, B. B. (2017). *IUCrJ* **4**, 476–485. 10.1107/S2052252517005553 PMC5571810 28875034

[bb8] Förster, A., Brandstetter, S. & Schulze-Briese, C. (2019). *Philos. Trans. R. Soc. A* **377**, 20180241. 10.1098/rsta.2018.0241 PMC6501887 31030653

[bb9] Giacovazzo, C., Monaco, H. L., Artioli, G., Viterbo, D., Milanesio, M., Gilli, G., Gilli, P., Zanotti, G., Ferraris, G. & Catti, M. (2011). *Fundamentals of Crystallography.* Oxford University Press.

[bb10] Guérin, L., Yoshida, T., Zatterin, E., Simonov, A., Chernyshov, D., Iguchi, H., Toudic, B., Takaishi, S. & Yamashita, M. (2022). *ChemPhysChem* **23**, e202100857. 10.1002/cphc.202100857 35083834

[bb11] Hansen, N. K. & Coppens, P. (1978). *Acta Cryst.* A**34**, 909–921.

[bb12] Harris, C. R., Millman, K. J., van der Walt, S. J., Gommers, R., Virtanen, P., Cournapeau, D., Wieser, E., Taylor, J., Berg, S., Smith, N. J., Kern, R., Picus, M., Hoyer, S., van Kerkwijk, M. H., Brett, M., Haldane, A., del Río, J. F., Wiebe, M., Peterson, P., Gérard-Marchant, P., Sheppard, K., Reddy, T., Weckesser, W., Abbasi, H., Gohlke, C. & Oliphant, T. E. (2020). *Nature* **585**, 357–362. 10.1038/s41586-020-2649-2 PMC7759461 32939066

[bb13] Hirshfeld, F. L. (1977). *Isr. J. Chem.* **16**, 226–229.

[bb14] Holm, K. A. U., Roth, N., Zeuthen, C. M., Tolborg, K., Feidenhans’l, A. A. & Iversen, B. B. (2020). *Phys. Rev. B* **102**, 024112.

[bb15] Jayatilaka, D. & Dittrich, B. (2008). *Acta Cryst.* A**64**, 383–393. 10.1107/S0108767308005709 18421128

[bb17] Jha, K. K., Kleemiss, F., Chodkiewicz, M. L. & Dominiak, P. M. (2023). *J. Appl. Cryst.* **56**, 116–127. 10.1107/S1600576722010883 PMC9901929 36777135

[bb18] Kabsch, W. (1988*a*). *J. Appl. Cryst.* **21**, 67–72.

[bb19] Kabsch, W. (1988*b*). *J. Appl. Cryst.* **21**, 916–924.

[bb20] Kabsch, W. (1993). *J. Appl. Cryst.* **26**, 795–800.

[bb21] Kabsch, W. (2010). *Acta Cryst.* D**66**, 125–132. 10.1107/S0907444909047337 PMC2815665 20124692

[bb22] Koch, R. J., Roth, N., Liu, Y., Ivashko, O., Dippel, A.-C., Petrovic, C., Iversen, B. B., **v**, Zimmermann, M. & Bozin, E. S. (2021). *Acta Cryst.* A**77**, 611–636. 10.1107/S2053273321010159 34726636

[bb23] Meekel, E. G., Schmidt, E. M., Cameron, L. J., Dharma, A. D., Windsor, H. J., Duyker, S. G., Minelli, A., Pope, T., Lepore, G. O., Slater, B., Kepert, C. J. & Goodwin, A. L. (2023). *Science* **379**, 357–361. 10.1126/science.ade5239 36701437

[bb24] Momma, K. & Izumi, F. (2011). *J. Appl. Cryst.* **44**, 1272–1276.

[bb25] Pauling, L. (1935). *J. Am. Chem. Soc.* **57**, 2680–2684.

[bb26] Pichon-Pesme, V., Lecomte, C. & Lachekar, H. (1995). *J. Phys. Chem.* **99**, 6242–6250.

[bb27] Poppe, R., Roth, N., Neder, R. B., Palatinus, L., Iversen, B. B. & Hadermann, J. (2024). *IUCrJ* **11**, 82–91. 10.1107/S2052252523010254 PMC10833392 38096038

[bb28] Roth, N., Beyer, J., Fischer, K. F. F., Xia, K., Zhu, T. & Iversen, B. B. (2021). *IUCrJ* **8**, 695–702. 10.1107/S2052252521005479 PMC8256708 34258017

[bb29] Roth, N. & Goodwin, A. L. (2023). *Nat. Commun.* **14**, 4328. 10.1038/s41467-023-40063-w PMC10356831 37468516

[bb30] Roth, N. & Iversen, B. B. (2019). *Acta Cryst.* A**75**, 465–473. 10.1107/S2053273319004820 31041902

[bb32] Sangiorgio, B., Bozin, E. S., Malliakas, C. D., Fechner, M., Simonov, A., Kanatzidis, M. G., Billinge, S. J. L., Spaldin, N. A. & Weber, T. (2018). *Phys. Rev. Mater.* **2**, 085402.

[bb33] Schmidt, E. M., Klar, P. B., Krysiak, Y., Svora, P., Goodwin, A. L. & Palatinus, L. (2023). *Nat. Commun.* **14**, 6512. 10.1038/s41467-023-41934-y PMC10579245 37845256

[bb34] Schmidt, E. M., Neder, R. B., Martin, J. D., Minelli, A., Lemée, M.-H. & Goodwin, A. L. (2023). *Acta Cryst.* B**79**, 138–147. 10.1107/S205252062300121X PMC10088481 36920878

[bb35] Sears, J., Shen, Y., Krogstad, M. J., Miao, H., Yan, J., Kim, S., He, W., Bozin, E. S., Robinson, I. K., Osborn, R., Rosenkranz, S., Kim, Y.-J. & Dean, M. P. M. (2023). *Phys. Rev. B* **108**, 144419.

[bb36] Simonov, A., De Baerdemaeker, T., Boström, H. L. B., Ríos Gómez, M. L., Gray, H. J., Chernyshov, D., Bosak, A., Bürgi, H.-B. & Goodwin, A. L. (2020). *Nature* **578**, 256–260. 10.1038/s41586-020-1980-y PMC7025896 32051599

[bb37] Simonov, A. & Goodwin, A. L. (2020). *Nat. Rev. Chem.* **4**, 657–673. 10.1038/s41570-020-00228-3 37127977

[bb38] Simonov, A., Weber, T. & Steurer, W. (2014). *J. Appl. Cryst.* **47**, 2011–2018.

[bb39] Snyder, G. J. & Toberer, E. S. (2008). *Nat. Mater.* **7**, 105–114. 10.1038/nmat2090 18219332

[bb40] Stewart, R. F. (1976). *Acta Cryst.* A**32**, 565–574.

[bb41] Støckler, K. A. H., Roth, N., Feidenhans’l, A. A., Takahashi, S., Nishibori, E. & Iversen, B. B. (2024). *Phys. Rev. Mater.* **8**, 034409.

[bb42] Støckler, K. A. H., Roth, N., Grønbech, T. B. E. & Iversen, B. B. (2022). *IUCrJ* **9**, 523–532. 10.1107/S2052252522006315 PMC9252160 35844472

[bb43] Støckler, K. A. H., Zhang, J. & Brummerstedt Iversen, B. (2024). *Chem. A Eur. J.* **30**, e202402001. 10.1002/chem.202402001 38924189

[bb44] Urban, P., Simonov, A., Weber, T. & Oeckler, O. (2015). *J. Appl. Cryst.* **48**, 200–211.

[bb45] Volkov, A., Li, X., Koritsanszky, T. S. & Coppens, P. (2004). *J. Phys. Chem. A* **108**, 4283–4300.

[bb46] Vosegaard, E. S., Ahlburg, J. V., Krause, L. & Iversen, B. B. (2023). *Acta Cryst.* B**79**, 380–391. 10.1107/S2052520623006625 PMC10552600 37669152

[bb47] Weber, T. & Simonov, A. (2012). *Z. Kristallogr.* **227**, 238–247.

[bb48] Weller, M., Overton, T., Rourke, J. & Armstrong, F. (2018). *Inorganic Chemistry*, 7th ed. Oxford University Press.

[bb49] Willis, B. M. T. & Pryor, A. W. (1975). *Thermal Vibrations in Crystallography.* Cambridge University Press.

[bb50] Zhang, J., Roth, N., Tolborg, K., Takahashi, S., Song, L., Bondesgaard, M., Nishibori, E. & Iversen, B. B. (2021). *Nat. Commun.* **12**, 6709. 10.1038/s41467-021-27007-y PMC8602660 34795243

