# Supplementary material for: Benchmarking 3D-ΔPDF analysis using in-house X-ray sources
Source: Acta Crystallogr A Found Adv. 2025 May 19;81(Pt 4):254–68. doi: 10.1107/S2053273325003389 (PMC12207916; doi:10.1107/S2053273325003389)
Supplement: Supplementary file 1 [file a-81-00254-sup1.pdf]

FOUNDATIONS
ADVANCES

Volume 81 (2025)

Supporting information for article:

Benchmarking 3D-ΔPDF analysis using in-house X-ray sources

Karl O. R. Juul, Kristoffer A. H. Støckler and Bo B. Iversen

Contents

Effects of fluorescence.....2

Measurement strategies.....4

Remaining interlayer assignments in Cu_2Se.....9

The effect of instrumental parameters on the Fourier transform.....11

Construction of integration boxes.....12

Effects of resolution.....12

Effects of fluorescence

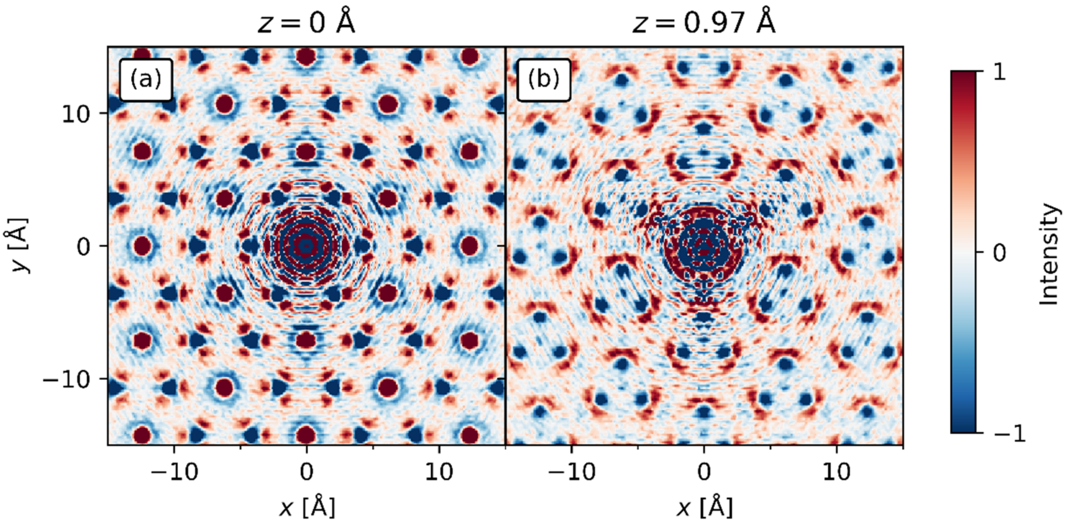

Figure S1. In-house measurements on $Cu_{1.95}Se$ identical to those in the main text, but without an energy threshold on the detector. The noise arises from fluorescence due to the K-edge of Se being close to the used X-ray energy. a) shows the $z = 0$ Å layer while b) shows the $z = 0.97$ Å layer. In both layers, the noise extends much further out in direct space and obscures some features close to the origin while feature further away become distorted and difficult to pinpoint and interpret.

Making the $Cu_{1.95}Se$ in-house measurements in an identical manner to that described in the main text, but without an energy threshold on the detector yielded a very noisy 3D-ΔPDF as shown in Fig. S1 in the $z = 0$ Å and $z = 0.97$ Å planes (corresponding to the layers shown in the main text). The noise arises from fluorescence effects due to the K-edge of Se being close to the used X-ray energy.
As fluorescence is polarization independent, application of polarization correction to fluorescence leads to an artificially increased background at high q (see fig. S2). As such, it is important to aim for avoiding fluorescence within the measurements and energy discrimination is thus preferable when possible.

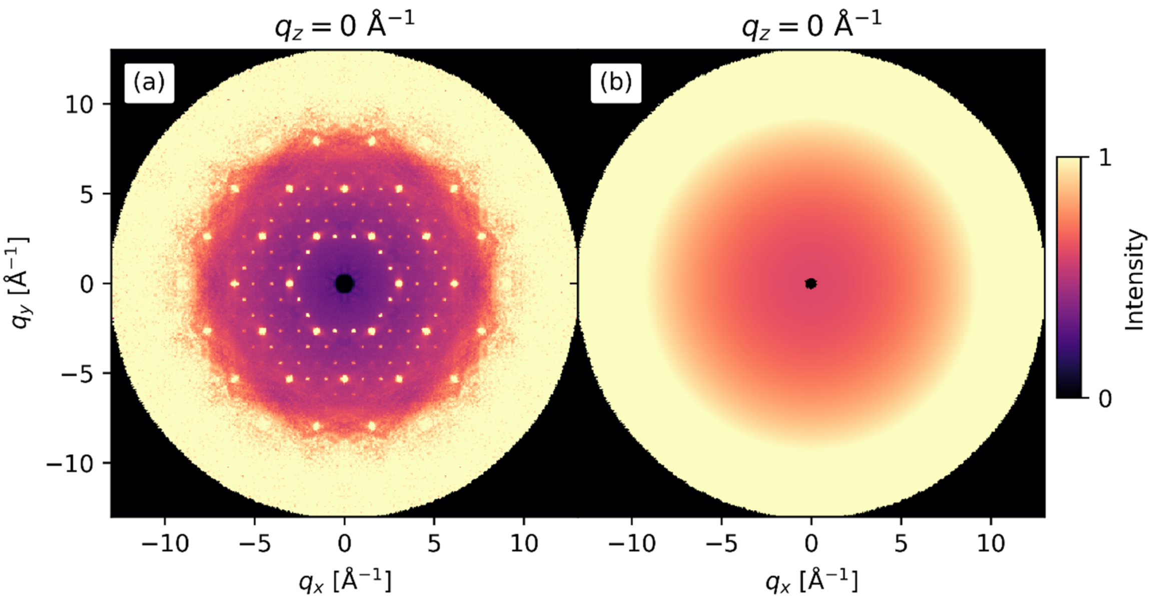

Figure S2. a) The symmetrized scattering pattern from the non-energy discriminated in-house measurement of $Cu_{1.95}Se$. b) Reconstruction of simulated isotropic fluorescence with applied polarization correction. Both a) and b) have been scaled to highlight the background. As is observed, the background in a) has the same form as the polarization corrected fluorescence in b) giving an artificially high background at high *q*.

Measurement strategies

Table S1. Measurement strategy for Cu_2Se diffuse scattering on in-house diffractometer.

ω_{start} [°]	ω_{end} [°]	Width [°]	Time [s]	2θ [°]	κ [°]	ϕ [°]	#Images
-75.0	75.0	0.2	8.0	0.0	0.0	0.0	750
-30.0	120.0	0.2	8.0	45.0	0.0	0.0	750
-25.0	79.0	0.2	8.0	0.0	30.0	0.0	520
20.0	124.0	0.2	8.0	45.0	30.0	0.0	520

Table S2. Measurement strategy for Cu_2Se air scattering on in-house diffractometer.

ω_{start} [°]	ω_{end} [°]	Width [°]	Time [s]	2θ [°]	κ [°]	ϕ [°]	#Images
-75.0	75.0	2.0	8.0	0.0	0.0	0.0	75
-30.0	120.0	2.0	8.0	45.0	0.0	0.0	75
-25.0	79.0	2.0	8.0	0.0	30.0	0.0	52
20.0	124.0	2.0	8.0	45.0	30.0	0.0	52

Table S3. Measurement strategy for $Nb_{1-x}CoSb$ SC diffuse scattering on in-house diffractometer.

ω_{start} [°]	ω_{end} [°]	Width [°]	Time [s]	2θ [°]	κ [°]	ϕ [°]	#Images
-75.0	75.0	0.2	100.0	0.0	0.0	0.0	750
0.0	150.0	0.2	100.0	75.0	0.0	0.0	750
-40.0	110.0	0.2	100.0	35.0	0.0	0.0	750

Table S4. Measurement strategy for $Nb_{1-x}CoSb$ SC air scattering on in-house diffractometer.

ω_{start} [°]	ω_{end} [°]	Width [°]	Time [s]	2θ [°]	κ [°]	ϕ [°]	#Images
-75.0	75.0	10.0	100.0	0.0	0.0	0.0	15
0.0	150.0	10.0	100.0	75.0	0.0	0.0	15
-40.0	110.0	10.0	100.0	35.0	0.0	0.0	15

Table S5. Measurement strategy for $Nb_{1-x}CoSb$ Q diffuse scattering on in-house diffractometer.

ω_{start} [°]	ω_{end} [°]	Width [°]	Time [s]	2θ [°]	κ [°]	ϕ [°]	#Images
-75.0	75.0	0.2	171.0	0.0	0.0	0.0	750
-5.0	145.0	0.2	171.0	70.0	0.0	0.0	750

Table S6. Measurement strategy for $Nb_{1-x}CoSb$ Q air scattering on in-house diffractometer.

ω_{start} [°]	ω_{end} [°]	Width [°]	Time [s]	2θ [°]	κ [°]	ϕ [°]	#Images

-75.0	65.0	20.0	171.0	0.0	0.0	0.0	7
-5.0	135.0	20.0	171.0	70.0	0.0	0.0	7

Table S7. Measurement strategy for InTe diffuse scattering on in-house diffractometer.

ω_{start} [°]	ω_{end} [°]	Width [°]	Time [s]	2θ [°]	κ [°]	ϕ [°]	#Images
-75.0	75.0	0.2	230.0	0.0	0.0	0.0	750
-25.0	80.0	0.2	230.0	0.0	30.0	0.0	525
-20.0	70.0	0.2	230.0	0.0	60.0	0.0	450
-35.0	115.0	0.2	230.0	40.0	0.0	0.0	750
15.0	120.0	0.2	230.0	40.0	30.0	0.0	525
20.0	110.0	0.2	230.0	40.0	60.0	0.0	450

Table S8. Measurement strategy for InTe air scattering on in-house diffractometer.

ω_{start} [°]	ω_{end} [°]	Width [°]	Time [s]	2θ [°]	κ [°]	ϕ [°]	#Images
-75.0	75.0	5.0	230.0	0.0	0.0	0.0	30
-25.0	80.0	3.5	230.0	0.0	30.0	0.0	30
-20.0	70.0	3.0	230.0	0.0	60.0	0.0	30
-35.0	115.0	5.0	230.0	40.0	0.0	0.0	30
15.0	120.0	4.2	230.0	40.0	30.0	0.0	25
20.0	110.0	3.6	230.0	40.0	60.0	0.0	25

Table S9. Measurement strategy for InTe diffuse scattering at SPring-8, BL02B1. Detector distance was 130 mm and the incident wavelength was 0.26355 Å.

ω_{start} [°]	ω_{end} [°]	#Images	Time [s]	χ [°]	ϕ [°]	2θ [°]
0	180.0	900	1.0	0	0	0
0	180.0	900	1.0	0	0	-10.0
0	180.0	900	1.0	0	0	-20.0
0	180.0	900	1.0	0	0	-30.0
0	180.0	900	1.0	45.0	0	0
0	180.0	900	1.0	45.0	0	-10.0
0	180.0	900	1.0	45.0	0	-20.0
0	180.0	900	1.0	45.0	0	-30.0

Table S10. Measurement strategy for InTe air scattering at SPring-8, BL02B1. Detector distance was 130 mm and the incident wavelength was 0.26355 Å.

ω_{start} [°]	ω_{end} [°]	#Images	Time [s]	χ [°]	ϕ [°]	2θ [°]
0	20.0	100	1.0	0	0	0
0	20.0	100	1.0	0	0	-10.0
0	20.0	100	1.0	0	0	-20.0
0	20.0	100	1.0	0	0	-30.0
0	20.0	100	1.0	45.0	0	0
0	20.0	100	1.0	45.0	0	-10.0
0	20.0	100	1.0	45.0	0	-20.0
0	20.0	100	1.0	45.0	0	-30.0

Table S11. Measurement strategy for diffuse scattering on Cu_2Se at APS, 15-ID-D ($\omega = -180°$ for all measurements). Data with different in-plane detector offsets were measured to limit overlap between masked areas of the detector. Detector distance was 120 mm and the incident energy was 40 keV.

ϕ_{start} [°]	ϕ_{end} [°]	#Images	Time [s]	κ [°]	Horizontal offset [mm]	Vertical offset [mm]
0	360.0	3600	1.0	0	122	98
0	360.0	3600	1.0	0	130	90
0	360.0	3600	1.0	0	138	82

Table S12. Measurement strategy for air scattering for Cu_2Se at APS, 15-ID-D ($\omega = -180°$). Three runs with different in-plane detector offsets were measured to limit overlap between masked areas of the detector. Detector distance was 120 mm and the incident energy was 40 keV.

ϕ_{start} [°]	ϕ_{end} [°]	#Images	Time [s]	κ [°]	Horizontal offset [mm]	Vertical offset [mm]
0	10.0	100	1.0	0	122	98
0	10.0	100	1.0	0	130	90
0	10.0	100	1.0	0	138	82

Table S13. Measurement strategy for Half Heusler Q-0.84 #2 diffuse scattering at SPring-8, BL02B1. Detector distance was 130 mm and the incident wavelength was 0.245479 Å.

ω_{start} [°]	ω_{end} [°]	#Images	Time [s]	χ [°]	ϕ [°]	2θ [°]
0	180.0	900	2.0	0	0	0
0	180.0	900	2.0	0	0	-12.5
0	180.0	900	2.0	0	0	-25
0	180.0	900	2.0	45	0	0
0	180.0	900	2.0	45	0	-12.5
0	180.0	900	2.0	45	0	-25

Table S14. Measurement strategy for air scattering for Half Heusler Q-0.84 #2 at SPring-8, BL02B1. Detector distance was 130 mm and the incident wavelength was 0.245479 Å.

ω_{start} [°]	ω_{end} [°]	#Images	Time [s]	χ [°]	ϕ [°]	2θ [°]
0	18.0	90	2.0	0	0	0
0	18.0	90	2.0	0	0	-12.5
0	18.0	90	2.0	0	0	-25

Table S15. Measurement strategy for Half Heusler SC-0.81 diffuse scattering at SPring-8, BL02B1. Detector distance was 260 mm and the incident wavelength was 0.245479 Å.

ω_{start} [°]	ω_{end} [°]	#Images	Time [s]	χ [°]	ϕ [°]	2θ [°]
0	180.0	900	0.4	0	0	0
0	180.0	900	0.4	0	0	-10
0	180.0	900	0.4	0	0	-20
0	180.0	900	0.4	0	0	-30
0	180.0	900	0.4	45	0	0
0	180.0	900	0.4	45	0	-10
0	180.0	900	0.4	45	0	-20
0	180.0	900	0.4	45	0	-30

Table S16. Measurement strategy for air scattering for Half Heusler SC-0.81 at SPring-8, BL02B1. Detector distance was 260 mm and the incident wavelength was 0.245479 Å.

ω_{start} [°]	ω_{end} [°]	#Images	Time [s]	χ [°]	ϕ [°]	2θ [°]
0	18.0	90	0.4	0	0	0
0	18.0	90	0.4	0	0	-10
0	18.0	90	0.4	0	0	-20
0	18.0	90	0.4	0	0	-30

Remaining interlayer assignments in Cu_2Se

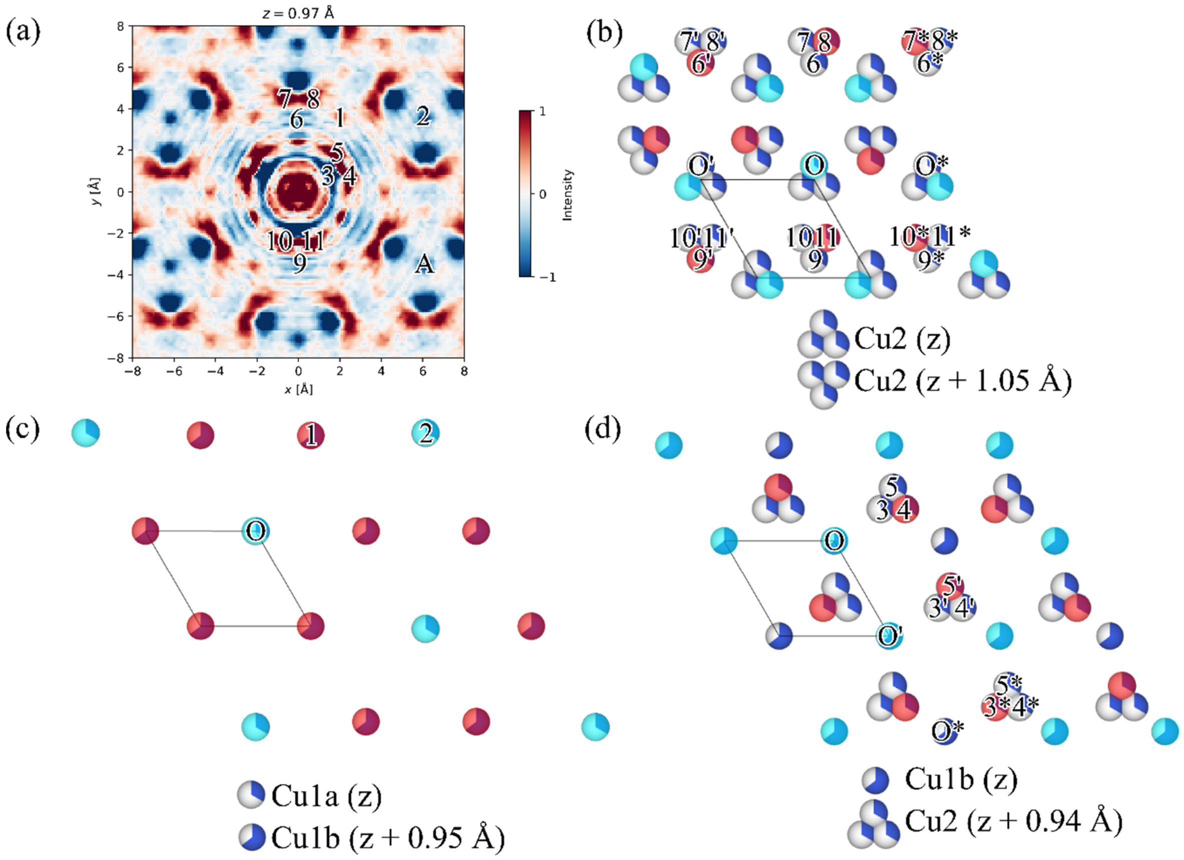

Figure S3. Inter-sublayer orderings between Cu-sublayers in $Cu_{1.95}Se$. All the inter-sublayer distances have a *z*-component close to $z = 0.97$ Å, which is why this is the slice of the 3D-ΔPDF, shown in (a), used for the analysis. Three different inter-sublayers are needed to completely assign the structural order. These are (b) Cu2-Cu2, (c) Cu1a-Cu1b, and (d) Cu1b-Cu2. The blue-tined atoms mark the occupied sites in the lower sublayer while the red-tinted atoms mark the occupied sites in the upper sublayer. Unmarked sites are unoccupied. The numbers on the atoms correspond to the location of the atom relative to the origin of the 3D-ΔPDF. Intensity is on an arbitrary scale.

As for the correlations between the Cu1b and Cu2-sublayers (Fig. S3(d)), the three Cu1b sites, marked O, O', and O* in Fig. S3(d), must be considered. The sites in the Cu2-sublayer are marked "3" through "5". These features are close to the noise region. However, they have higher intensity than the noise, and they can thus be considered as non-noise features. If this is not desired, the 2D order can be utilized and the same arrangement of features relative to the feature at "2" can be considered instead. Regardless, the positive features at "4" and "5" correspond to separating a vacancy-pair or an atom-pair (or both). This suggests an ordering like the one shown in Fig. S3(d). Roth & Iversen (2019) further supported this through equation (1), where the vector to "3" can separate either a trio of atom-vacancy pairs (like in the model shown) or a set of atom-vacancy, atom-atom, and vacancy-vacancy pairs. If the latter was

the case, the peak at “3” would have an amplitude of $\delta Z_{\mathrm{O}}\delta Z_{\mathrm{vac}} + \delta Z_{\mathrm{O}'}\delta Z_{\mathrm{occ}} + \delta Z_{\mathrm{O}^*}\delta Z_{\mathrm{vac}} = \frac{1}{3}\cdot\frac{-1}{3}Z_{\mathrm{Cu}}^2 + \frac{1}{3}\cdot\frac{2}{3}Z_{\mathrm{Cu}}^2 + \frac{-2}{3}\cdot\frac{-1}{3}Z_{\mathrm{Cu}}^2 = \frac{1}{3}Z_{\mathrm{Cu}}^2$. Since the corresponding experimental 3D-ΔPDF signal is negative, the vector to “3” cannot separate even a single pair of occupied atoms, which supports the model shown.

The Cu2-Cu2 inter-sublayer ordering (Fig. S3(b)) reveals little new information regarding the difference between the synchrotron and in-house 3D-ΔPDFs. However, the features marked “6” and “9” in Fig. S3 are much more difficult to see in the in-house 3D-ΔPDF due to their weak nature and the surrounding noise. The smoother background and much reduced noise on the synchrotron 3D-ΔPDF make the weak features more apparent as independent features. In a case, where no synchrotron data had been available, these might have been deemed too weak and affected by Fourier ripples to be included in the analysis. However, including them in the analysis allows determining the Cu2-Cu2 inter-sublayer ordering as Roth & Iversen (Roth & Iversen, 2019), who found the ordering shown in Fig. S3(b) and its mirror image to be present in the structure. Assigning atoms in the lower Cu2-sublayer and choosing “O” to be the only occupied origin, an ordering like the one shown in Fig. S3(b) can be proposed (Roth & Iversen, 2019). In the same manner as for the Cu1b-Cu2 ordering, one can find the vectors to “6” and “9”, respectively, to separate three atom-vacancy pairs by using equation (1). If they respectively separate at least one pair of occupied sites, their amplitudes would be proportional to $\delta Z_{\mathrm{O}}\delta Z_{\mathrm{occ}} + 2\delta Z_{\mathrm{origin,vac}}\delta Z_{\mathrm{vac}} = \left(1-\frac{1}{3}\right)\left(1-\frac{1}{3}\right)Z_{\mathrm{Cu}}^2 + 2\left(0-\frac{1}{3}\right)\left(0-\frac{1}{3}\right)Z_{\mathrm{Cu}}^2 = \frac{2}{3}Z_{\mathrm{Cu}}^2$. Thus, they must separate three atom-vacancy pairs.

The effect of instrumental parameters on the Fourier transform

In this section, we examine two effects of instrumental parameters on the resulting 3D-ΔPDF by considering their effects on the general Fourier transform. Firstly, an isotropic broadening which is caused by the instrumental resolution function. The second is a radial broadening, which is caused by the use of non-monochromatic beam (e.g., the use of Kα_1 and Kα_2).

An isotropic and uniform broadening of all features in reciprocal space can be caused by the instrumental resolution function (IRF). The final scattering signal will be a convolution of the non-broadened signal with the IRF. The convolution theorem states that for two functions, the Fourier transform of a convolution of two functions is the multiplication of their respective Fourier transforms. That is, for two functions, f and g, $\mathcal{F}[f * g] = \sqrt{2\pi}\mathcal{F}[f]\mathcal{F}[g]$ (Boggess & Narcowich, 2009). Assuming that the IRF has a Gaussian peak shape, its Fourier transform will also be a Gaussian. Thus, this will lead to a dampening of the 3D-ΔPDF at high r (Weber & Simonov, 2012).

However, the broadening caused by the use of Kα_1 and Kα_2 is not isotropic. The reason being that this corresponds to a distribution of wavelengths (Weber & Simonov, 2012). The difference between them is a scaling of the q coordinate. Consider a monochromatic beam of wavelength, λ, then $\boldsymbol{q} = \frac{2\pi}{\lambda}(\boldsymbol{s_f} - \boldsymbol{s_i})$ where $\boldsymbol{s_i}$ and $\boldsymbol{s_f}$ are unit vectors in the direction of the incoming and diffracted beam, respectively. Consider a non-monochromatic beam consisting of wavelengths λ_j each with its own weight w_j. For each of these, $\boldsymbol{q}$, becomes $\boldsymbol{q}_j = \frac{2\pi}{\lambda_j}(\boldsymbol{s_f} - \boldsymbol{s_i}) = \frac{2\pi}{c_j\lambda}(\boldsymbol{s_f} - \boldsymbol{s_i}) = \frac{1}{c_j}\boldsymbol{q}$ where c_j is a scaling constant close to unity. For the radially broadened scattering signal, $T'(\boldsymbol{q}) = \sum w_j T(\boldsymbol{q}_j)$, the Fourier transform can be derived using basic properties of the Fourier transform (Boggess & Narcowich, 2009)

$$\mathcal{F}[T'(\boldsymbol{q})](\boldsymbol{r}) = \mathcal{F}\left[\sum w_j T(\boldsymbol{q}_j)\right](\boldsymbol{r}) = \sum w_j \mathcal{F}\left[T\left(\frac{\boldsymbol{q}}{c_j}\right)\right](\boldsymbol{r}) = \sum c_j w_j \mathcal{F}[T(\boldsymbol{q})](c_j\boldsymbol{r}).$$

The notation $\mathcal{F}[T'(\boldsymbol{q})](\boldsymbol{r})$ means the Fourier transform of $T'(\boldsymbol{q})$ evaluated at $\boldsymbol{r}$. This shows that the Fourier transform of a radially broadened scattering signal at $\boldsymbol{r}$ is a sum of the non-broadened scattering signal with radially scaled coordinates.

Simulations of both effects can be seen in Weber & Simonov (2012).

Construction of integration boxes

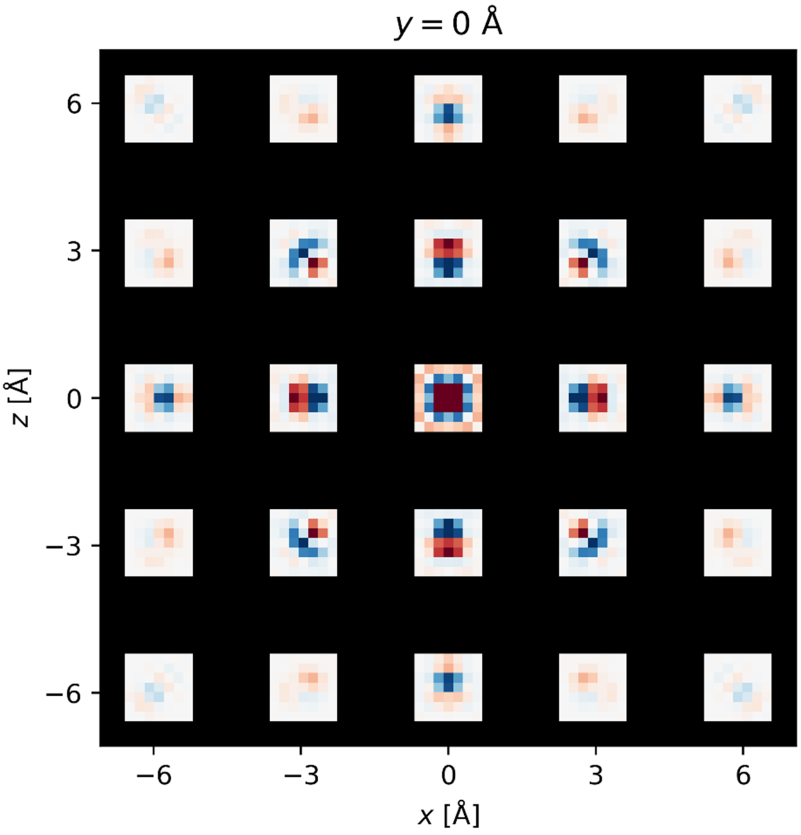

Figure S4. The 7 x 7 x 7 integration boxes used for the in-house sample. Black areas are masked to highlight the peak boxes, although the masked areas were also integrated in their own boxes. Integrals are calculated as the sum of each isolated volume.

Integration of the peaks in the 3D-ΔPDF was achieved using box integration. To construct the boxes, a box of size of *n* x *n* x *n* voxels around the peak centre (half-integer values of the unit cell axes as shown in Fig. S4) was constructed and the voxels within each volume were summed to give the final integrals. The masked volumes were given an integral of zero in the integrated 3D-ΔPDF. Each box was then assigned a single voxel in the integrated 3D-ΔPDF.

Effects of resolution

For the purpose of showing the capabilities of the sources compared in the main text, the synchrotron data has been reconstructed and transformed on a larger grid with higher resolution than the in-house data. To see instrumental effects and compare the measurement on the same resolution and grid size,

the synchrotron data has here been reconstructed and a 3D-ΔPDF made with the same grid size (601 x 601 x 601), reciprocal space extent (16 $Å^{-1}$), and all other parameters identical to the in-house reconstruction. This will be referred to as the low-resolution synchrotron data reduction, although it should be stressed that it is reconstructed using the exact same data as the one shown in the main text. The total scattering patterns are shown in Fig. S5.

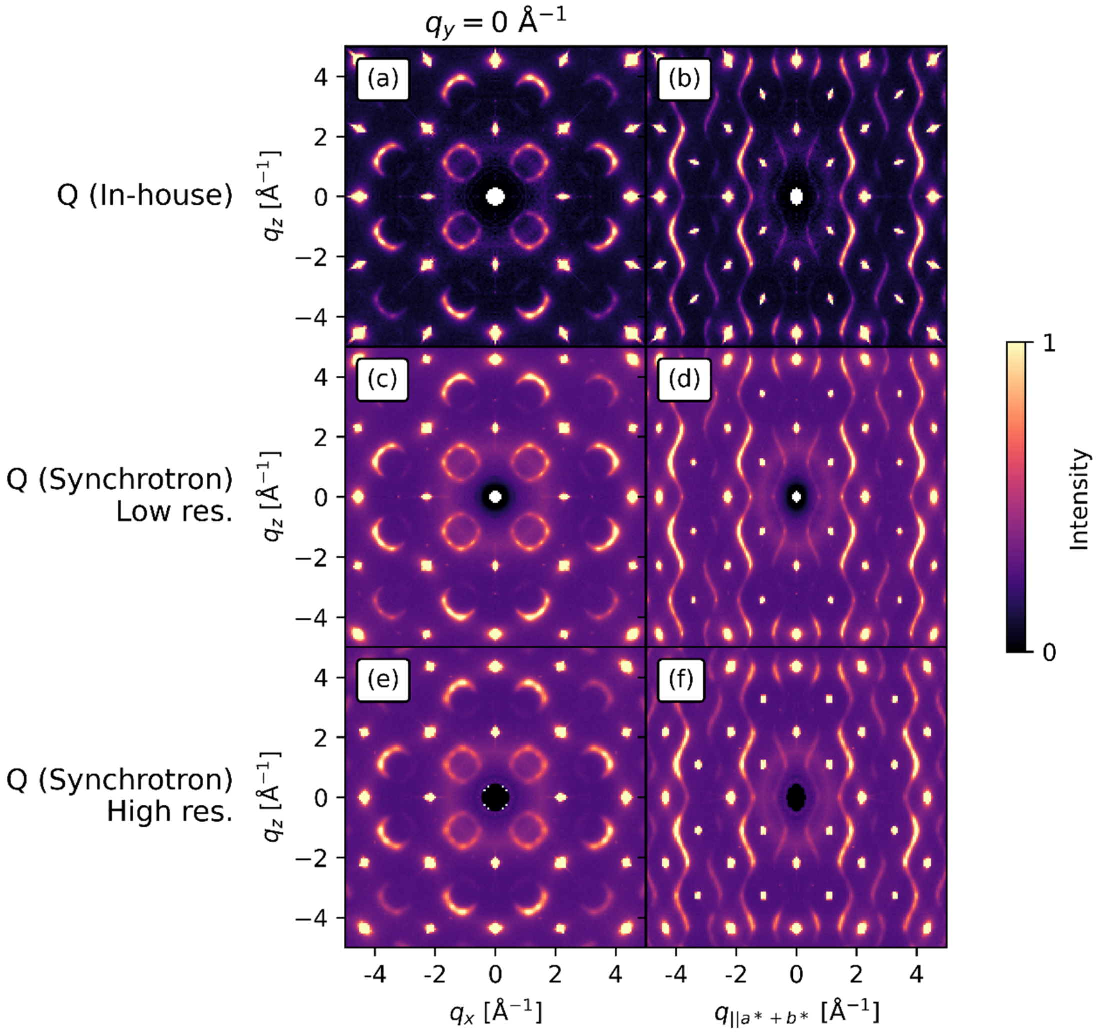

Figure S5. The total scattering patterns in the $q_y = 0$ Å^{-1} plane (left column) and the plane perpendicular to $\boldsymbol{a}^* - \boldsymbol{b}^*$ (right column) for (a)-(b) the quenched sample in-house shown in the main text, (c)-(d) quenched sample from synchrotron using the same data reduction parameters as the in-house data in (a), and (e)-(f) the synchrotron data shown in the main text given here for easier comparison. All plots are on arbitrary scale.

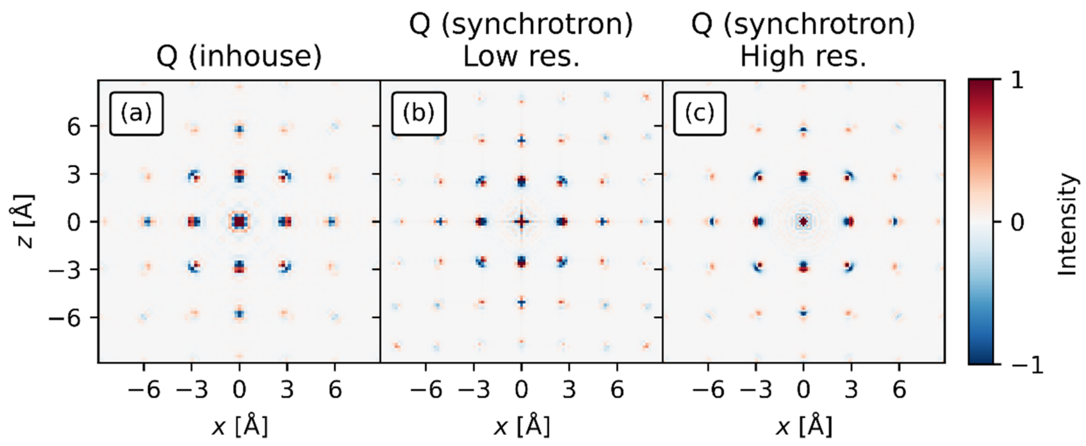

Figure S6. The resulting 3D-ΔPDF in the $y = 0$ Å plane from the (a) quenched sample inhouse, (b) low-resolution synchrotron, and (c) high-resolution synchrotron. The low-resolution synchrotron 3D-ΔPDF has been treated with the exact same parameters as the inhouse data in (a). All plots are on an arbitrary scale. (a) and (c) are shown in the main text and are provided here for easier comparison.

The resulting 3D-ΔPDF from the low-resolution synchrotron data reduction can be seen in Fig. S6 along with the 3D-ΔPDF from the in-house and the synchrotron shown in the main text. The low-resolution synchrotron 3D-ΔPDF is visually very similar to the other two. Due to the radial broadening in the in-house data, some of the peaks appear wider in the inhouse 3D-ΔPDF (Fig. S6(a)) compared to the low-resolution synchrotron 3D-ΔPDF (Fig. S6(b)). The low-resolution synchrotron 3D-ΔPDF still has less noise around the origin compared to the in-house data and while it does not have the same round shape as the high-resolution data, it still shows the same feature, the positive circle surrounded by a negative ring. Thus, it is clear that the resolution from the in-house data is sufficient and that the radial broadening from the in-house source is not problematic. However, it should be stressed that the resolution obtained from the synchrotron measurements is (often) much higher than the in-house and using the full capabilities of the synchrotron is preferrable.

Boggess, A. & Narcowich, F. J. (2009). *A First Course in Wavelets with Fourier Analysis*, 2nd ed. John Wiley & Sons.
Roth, N. & Iversen, B. B. (2019). *Acta Cryst.* **A75**, 465-473.
Weber, T. & Simonov, A. (2012). *Zeitschrift für Kristallographie* **227**, 238-247.
